# Phenyllactic Acid Suppresses Growth and Induces Coordinated Changes in PSII Photochemistry and Energy Dissipation in the Filamentous Cyanobacterium *Dolichospermum* sp. FACHB-82

**DOI:** 10.3390/microorganisms14102217

**Published:** 2026-10-02

**Authors:** Jie Cheng, Long Tan, Xiu Zhang, Zhenxia Zhu, Jiashun Li, Chaobo Zhang, Kaidian Zhang, Yongcheng Ding

**Affiliations:** 1 Shandong Key Laboratory of Applied Technology for Protein and Peptide Drugs, School of Pharmaceutical Sciences and Food Engineering, Liaocheng University, Liaocheng 252000, China; chengjie@lcu.edu.cn (J.C.); tldyp6789@163.com (L.T.); 15216501768@163.com (X.Z.); 17661802272@163.com (Z.Z.); zhangchaobo@lcu.edu.cn (C.Z.); 2 State Key Laboratory of Marine Resources Utilization in South China Sea, School of Marine Sciences, Hainan University, Haikou 570100, China; kzhang@hainanu.edu.cn; 3 Shanghai Academy of Environmental Sciences, Shanghai 200233, China

**Keywords:** phenyllactic acid, *Dolichospermum* sp. FACHB-82, harmful cyanobacterial blooms, photosynthetic response, chlorophyll *a* fluorescence rise kinetics

## Abstract

*Dolichospermum* sp. FACHB-82, a representative harmful bloom-forming cyanobacterium, has garnered widespread attention in freshwater ecosystems due to its high environmental adaptability and potential for rapid proliferation. Phenyllactic acid (PLA) is a structurally defined aromatic organic acid with known antimicrobial activity, but its inhibitory effects on FACHB-82 and their photosynthetic apparatus remain poorly understood. In this study, we investigated the concentration-dependent effects of PLA on biomass accumulation, photosynthetic pigment production and photosynthetic activity in FACHB-82. The findings revealed that PLA induced a concentration-dependent biphasic response on biomass accumulation and photosynthetic pigment synthesis of FACHB-82. Chlorophyll *a* fluorescence analyses revealed that high-concentration PLA exposure disrupted functions of PSII at multiple levels, including reduced PSII performance, enhanced Q_A_^−^ accumulation, accelerated reaction center closure, decreased energetic connectivity, impaired donor side function, and restricted downstream electron transport. Although NPQ was significantly upregulated under high PLA stress, dark-recovery analysis demonstrated that the increased non-photochemical energy dissipation was accompanied by incomplete recovery of PSII photochemical efficiency. Our finding, for the first time, provides physiological insight into the anticyanobacterial activity of PLA for the control of FACHB-82 growth, thus offering a theoretical basis for the control of cyanobacterial blooms.

## 1. Introduction

Eutrophication-driven cyanobacterial blooms remain a persistent challenge for freshwater ecosystem restoration and water quality management. Excessive inputs of nitrogen and phosphorus promote the proliferation of bloom-forming cyanobacteria, leading to reduced water transparency, oxygen depletion, altered aquatic community structure and deterioration of ecosystem functions [1,2]. In addition, many bloom-forming cyanobacteria produce cyanotoxins and other bioactive secondary metabolites that can cause lethal or sublethal effects in aquatic organisms and pose risks to drinking-water safety and public health [2,3,4]. Consequently, controlling harmful cyanobacterial blooms has become an important goal in the management of eutrophic freshwater systems.

While reducing external nutrient inputs is a fundamental strategy for the long-term mitigation of algal blooms, ecosystem recovery is often delayed due to the continuous release of internal phosphorus loads. Furthermore, favorable hydrological and climatic conditions, coupled with the physiological adaptability of cyanobacteria, can sustain bloom persistence [2,5,6]. To achieve rapid control, common interventions such as physical removal, chemical algicides, or biomanipulation are frequently employed for the short-term suppression of algal biomass. However, the widespread implementation of these methods is significantly hindered by high operational costs, transient efficacy, and the risks of secondary pollution, including the sudden release of toxins and adverse impacts on non-target species [7,8,9]. These challenges have prompted the scientific community to seek more selective and ecologically compatible alternatives for the suppression of harmful algal proliferations.

Allelopathy encompasses the advantageous or harmful biochemical interactions facilitated by secondary metabolites released by plants, algae, bacteria, or fungi, which can affect the growth, development, and physiological performance of neighboring organisms [10,11]. Consequently, naturally derived allelochemicals have emerged as potential alternatives or supplements to conventional bloom-control methods. In aquatic ecosystems, plants, microorganisms, and algae can release a diverse array of allelochemicals, including phenolic compounds, organic acids, fatty acids, alkaloids, and terpenoids. These compounds have the potential to regulate cyanobacterial growth by influencing cell membrane integrity, oxidative balance, nutrient uptake, pigment synthesis, and photosynthetic activity [12,13,14].

Low-molecular-weight aromatic organic acids, particularly phenolic acids, constitute a significant class of naturally occurring allelochemicals that have garnered substantial attention due to their environmental prevalence and extensive biological activity [15]. Various compounds within this class, including benzoic acid, salicylic acid, ferulic acid, caffeic acid, cinnamic acid, p-coumaric acid, and 3-hydroxycinnamic acid, have been documented to exert concentration-dependent regulatory effects on the proliferation of *Microcystis aeruginosa* [16,17]. These biphasic responses indicate that phenolic acids may function not only as toxic stressors but also as regulatory molecules that modulate the physiological status of cyanobacteria based on exposure levels. Furthermore, previous research has demonstrated that phenolic acids can disrupt multiple cellular processes in cyanobacteria, such as membrane integrity, oxidative balance, pigment metabolism, and photosynthetic performance [16,17,18]. Photosynthesis is particularly regarded as a primary physiological target of allelochemical stress, given that cyanobacterial growth is intrinsically reliant on the effective conversion of light energy into chemical energy. Alterations in chlorophyll content and chlorophyll fluorescence parameters have been documented following exposure to phenolic acids, suggesting that these compounds may disrupt photosynthetic electron transport and energy conversion processes. However, the majority of existing research has concentrated on conventional plant-derived phenolic acids and unicellular bloom-forming cyanobacteria, such as *M. aeruginosa*. In contrast, there is a paucity of information concerning the responses of filamentous cyanobacteria to structurally related, non-phenolic aromatic organic acids. Consequently, examining the effects of aromatic organic acids on filamentous cyanobacteria could yield further insights into their anticyanobacterial potential and mechanisms of action.

Phenyllactic acid (PLA) is an aromatic organic acid characterized by the presence of a benzene ring and a carboxyl functional group, distinguishing it from common phenolic acids due to the absence of a phenolic hydroxyl group on its aromatic moiety. In recent years, PLA has garnered significant research interest owing to its broad-spectrum antibacterial and antifungal properties [19,20]. More broadly, naturally derived bioactive preparations have also been reported to exhibit antimicrobial and antibiofilm activities against bacterial pathogens [21,22]. Although these studies do not directly concern PLA or cyanobacteria, they provide broader context for the antimicrobial potential of naturally derived chemical systems. Given its natural origin, well-defined chemical structure, and potent antimicrobial efficacy, PLA has been investigated for applications in food preservation, microbial management, and bio-based antimicrobial applications [19]. These diverse biological activities suggest that PLA may also serve as a promising naturally derived agent for regulating algal growth. Notably, PLA produced by *Leuconostoc mesenteroides* DH has been reported to contribute to the elimination of *M. aeruginosa*, inducing cellular damage, oxidative stress, and photosynthetic impairment, thereby indicating its potential involvement in anticyanobacterial activity [23]. However, the effects of purified PLA on filamentous cyanobacteria remain insufficiently understood.

Photosynthesis is one of the most vulnerable physiological processes in cyanobacteria when subjected to environmental stress. Photosystem II (PSII) is particularly susceptible to stress-induced damage. Given that the proliferation of cyanobacteria fundamentally relies on light-energy capture, excitation-energy conversion, and photosynthetic electron transport, any compromise to the integrity of PSII can swiftly disrupt cellular energy homeostasis and hinder growth performance. Chlorophyll *a* fluorescence analysis offers a rapid and non-invasive method for assessing PSII functionality in photosynthetic organisms. The fast fluorescence induction curve, known as the OJIP transient, illustrates the sequential reduction of electron carriers from the initial O step to the maximal P step. Through the JIP-test, OJIP analysis provides quantitative insights into PSII reaction-center activity, excitation energy trapping, electron transport beyond Q_A_, and energy dissipation [24,25,26]. Additionally, relative fluorescence difference kinetics, including L-band and K-band analyses, can further elucidate changes in energetic connectivity among PSII units and the donor-side function of PSII, respectively. And the I–P phase of the fluorescence transient can provide additional information on electron transport processes downstream of PSII [27].

Non-photochemical quenching (NPQ) analysis provides complementary information on the balance between photochemical energy use and excess-energy dissipation. Parameters such as effective quantum yield (QY), photochemical quenching (Qp) and NPQ can be used to evaluate PSII photochemical efficiency, reaction-center openness and the capacity for non-photochemical energy dissipation during light induction and dark recovery [28,29]. Therefore, combining OJIP/JIP-test analysis with NPQ measurements can provide a more integrated view of how PLA affects PSII energy conversion, electron transport and photoprotective responses in cyanobacteria. Based on these considerations, we used *Dolichospermum* sp. FACHB-82 as a filamentous cyanobacterial model to evaluate the concentration-dependent effects of PLA and to clarify whether PLA-induced inhibition is associated with PSII dysfunction. Specifically, we investigated the effects of PLA on biomass accumulation, photosynthetic pigment production, OJIP fluorescence transients, JIP-test parameters, relative fluorescence difference kinetics, IP-phase dynamics and NPQ responses. This study aims to characterize the photosynthetic physiological responses of filamentous cyanobacteria to PLA and to assess its potential as a candidate anticyanobacterial compound.

## 2. Materials and Methods

### 2.1. Algal Strain and Culture Conditions

*Dolichospermum* sp. FACHB-82 was obtained from the Freshwater Algae Culture Bank at the Institute of Hydrobiology (FACHB), Chinese Academy of Sciences, Wuhan, China, and was maintained under axenic conditions. The strain was cultured in BG11 medium (Product No. HB8793; Qingdao Hope Bio-Technology Co., Ltd., Qingdao, China) under controlled laboratory conditions at 25 °C, with a 14:10 h light/dark photoperiod. Illumination was provided by an LED panel light, and the cultivation irradiance at the culture surface was 30 μmol photons m^−2^ s^−1^. The cultures were incubated on a rotary shaker at 120 rpm to ensure homogeneous growth, and cells at the logarithmic growth phase were collected for subsequent PLA exposure experiments.

### 2.2. Preparation of PLA Stock Solution and Exposure Design

D-phenyllactic acid (PLA, ≥98%, Product No. S67778-5g, CAS No. 7326-19-4) was purchased from Shanghai Yuanye Bio-Technology Co., Ltd. (Shanghai, China), and the PLA stock solution of 10.0 g·L^−1^ was prepared by dissolving 0.1 g PLA in 10.0 mL BG11 medium. Then, the stock solution was further diluted with BG11 medium to achieve final PLA concentrations of 0.0, 100.0, 150.0 and 200.0 mg·L^−1^. The stated PLA concentrations represent nominal exposure concentrations, and the concentrations of PLA were not monitored during the 48 h exposure period. Meanwhile, the pH of the exposure medium was not adjusted after PLA addition, and pH was not monitored during the exposure experiments. For exposure experiments, FACHB-82 cultures were treated with different concentrations of PLA and incubated under the same culture conditions as described in Section 2.1. Algal biomass was determined at 0, 24, and 48 h after PLA exposure, and photosynthetic pigment and chlorophyll fluorescence analyses, including OJIP and NPQ measurements, were performed after 48 h of exposure. Each treatment was performed with three biological replicates. To assess possible optical interference from PLA, we scanned cell-free BG11 solutions containing 100 or 200 mg·L^−1^ PLA from 320 to 750 nm on a UV–Vis spectrophotometer (Shanghai Youke Instrument Co., Ltd., Shanghai, China), using blank BG11 medium as the reference. Two wavelength regions were of interest: 455 nm, the excitation wavelength for chlorophyll fluorescence, and 680 nm, where OD-based biomass estimation is performed. Absorbance at both wavelengths was close to zero under the tested conditions (Figure S1).

### 2.3. Determination of Algal Biomass

A linear relationship between algal cells and absorbance values (OD_680_) was first established to quickly estimate the biomass of algal cells. Specifically, algal cells with different cell densities were measured at 680 nm using an UV–Vis spectrophotometer. Meanwhile, the corresponding cells were obtained by centrifugation (8000 rpm, 5 min) and washed twice with distilled water. The sediment was dried at 60 °C until the cell weight was constant, and the biomass was subsequently recorded. For each treatment, algal cultures were sampled at 0, 24 and 48 h after PLA exposure, and OD_680_ was measured. OD_680_-derived biomass concentration was estimated according to the calibration equation. The biomass productivity (mg·L^−1^·d^−1^) was calculated as P = (X_t_ − X_0_)/t, where P is biomass productivity, X_t_ is the biomass concentration at exposure time, X_0_ is the initial biomass concentration, and t is the exposure time. The specific growth rate (d^−1^) was measured as u = (lnXt − lnX_0_)/t, where X_t_ is the biomass concentration at exposure time, X_0_ is the initial biomass concentration, and t is the exposure time. The relative biomass change of PLA-treated cultures compared with the control was calculated as P′ = (X_treatment_ − X_control_) × 100/X_control_, where P′ is relative biomass change (%), and X_treatment_ and X_control_ represent the biomass concentrations of the PLA-treated and control groups at the same exposure time, respectively. Each treatment was performed with three biological replicates.

### 2.4. Photosynthetic Pigment Levels in Algal Cells

After 48 h of PLA exposure, photosynthetic pigments were extracted using 95% ethanol (Tianjin Fuyu Fine Chemical Co., Ltd., Tianjin, China). Briefly, 4.0 mL of algal culture was collected from each sample and centrifuged at 8000 rpm for 5 min. The supernatant was discarded, and the algal pellet was collected. Glass grinding beads (0.5 mm; Wuhan Servicebio Technology Co., Ltd., Wuhan, China; Cat. No. G0350-40G) and 95% ethanol were then added to the pellet, followed by cell disruption using a portable tissue grinder (TL-MINI-4, Toroivd, Jiangsu, China), and homogenization was performed for 30 s per cycle for five cycles. The disrupted cell suspension was kept in the dark for 24 h to ensure complete extraction of photosynthetic pigments. After extraction, the suspension was centrifuged at 12,000 rpm for 5 min, and the supernatant was collected for spectrophotometric analysis. The absorbance of the extract was measured at 665, 649 and 470 nm. The concentrations of chlorophyll *a* and carotenoids were calculated spectrophotometrically according to previously described ethanol-extraction methods [30]. Chlorophyll *a* concentration was calculated according to the following equation: Chl *a* = 13.7 × OD_665_ − 5.76 × OD_649_, and the carotenoid concentration was calculated using the following equation: Caro = (1000 × OD_470_ − 2.05 × Chl *a*)/245, where Chl *a* represents chlorophyll *a* content and Caro represents carotenoid content. Pigment titer was expressed as μg·mL^−1^, and pigment yields were calculated by normalizing pigment titer to algal biomass and expressed as μg·μg^−1^. Each treatment was analyzed with three biological replicates.

### 2.5. Chlorophyll a Fluorescence Transient Measurement

Chlorophyll *a* fluorescence transients were measured using a portable chlorophyll fluorometer (AquaPen-C AP110-C, Photon Systems Instruments, Drásov, Czech Republic). Before measurement, 4.0 mL of algal culture was collected from each treatment and diluted with fresh PLA-free BG11 medium for the same cell density (OD_680_ = 0.2) to minimize differences in cell density and sample turbidity among treatments, and no separate correction for PLA-specific optical absorption was applied. Then, 3.0 mL of the diluted algal suspension was transferred into a cuvette and dark-adapted for 30 min. After dark adaptation, fast chlorophyll *a* fluorescence induction kinetics, namely OJIP transients, were recorded using the OJIP protocol. The excitation light was set to blue light at 455 nm. The measuring flash pulse and saturating super pulse were set to 30% and 50%, respectively. The data for OJIP curves and JIP-test parameters were recorded and exported for subsequent analysis, and the specific meanings of chlorophyll fluorescence parameters are shown in Table S1.

### 2.6. OJIP-Test and Relative Fluorescence Analysis

OJIP fluorescence transients were analyzed using the JIP-test and relative fluorescence normalization methods. Fo was defined as the fluorescence intensity at 50 μs, F_K_ was defined as the fluorescence intensity at 300 μs, F_J_ was defined as the fluorescence intensity at 2 ms, F_I_ was defined as the fluorescence intensity at 30 ms, and Fm was defined as the maximal fluorescence intensity. Fv/Fm and Fv/Fo were calculated as (Fm − Fo)/Fm and (Fm − Fo)/Fo, respectively. The relative variable fluorescence was calculated as Vt = (F_t_ − Fo)/(Fm − Fo), and the difference kinetics were calculated as δVt = Vt_(treatment)_ − Vt_(control)_. The L-band, K-band, O–I phase and I–P phase were analyzed using W_OK_ = (F_t_ − Fo)/(F_K_ − Fo), W_OJ_ = (F_t_ − Fo)/(F_J_ − Fo), W_OI_ = (F_t_ − Fo)/(F_I_ − Fo) and W_IP_ = (F_t_ − F_I_)/(F_P_ − F_I_), respectively. The corresponding difference kinetics were calculated by subtracting the control curves from the treatment curves. The initial slope of the fluorescence rise was calculated as Mo = 4 × (F_K_ − Fo)/(Fm − Fo). The relative variable fluorescence at the J and I steps was calculated as V_J_ = (F_J_ − Fo)/(Fm − Fo) and V_I_ = (F_I_ − Fo)/(Fm − Fo), respectively. The active fraction of oxygen evolving complexes centers was estimated as OEC centers = [1 − (V_K_/V_J_)]_treatment_/[1 − (V_K_/V_J_)]_control_, and δ_Ro_ was calculated as (1 − V_I_)/(1 − V_J_). Other JIP-test parameters, including PIabs, ABS/RC, TRo/RC, ETo/RC, DIo/RC, ETo/TRo and ETo/ABS, were obtained from the AquaPen software or calculated by the authors from the exported fluorescence data using the equations described above.

### 2.7. Non-Photochemical Quenching Measurement

NPQ was measured using the built-in NPQ1 protocol of the AquaPen-C AP110-C fluorometer. Before measurement, algal suspensions were adjusted to the same cell density (OD_680_ = 0.2) with fresh BG11 medium to minimize differences in cell density and sample turbidity among treatments, and no separate correction for PLA-specific optical absorption was applied. Then, 3.0 mL of each suspension was transferred into a clean cuvette and dark-adapted for 30 min. After dark adaptation, the NPQ1 protocol was initiated. Minimal fluorescence in the dark-adapted state (Fo) was first recorded, followed by a saturating pulse to determine maximal fluorescence (Fm). The samples were then exposed to actinic light to induce light adaptation. Blue excitation light at 455 nm was used, with the measuring flash pulse and saturating super pulse set to 30% and 50%, respectively. The actinic light intensity was set to 30 μmol photons·m^−2^·s^−1^, corresponding approximately to the irradiance used for cultivation. Thus, the NPQ measurements were intended to characterize energy partitioning under the cultivation-light condition rather than the maximal NPQ capacity across a light-response range.

During the light induction phase, fluorescence parameters were recorded at L1, L2, L3, L4 and Lss, where L1–L4 represent sequential light-adapted measurement points and Lss represents the light-adapted steady state. After the actinic light was switched off, dark recovery parameters were recorded at D1, D2 and D3. QY, Qp, and NPQ were calculated automatically by the instrument software and exported using FluorPen software (version 1.1; Photon Systems Instruments, Drásov, Czech Republic) for subsequent analysis. Briefly, NPQ was calculated as (Fm − Fm′)/Fm′, where Fm represents the maximal fluorescence in the dark-adapted state, and Fm′ represents the maximal fluorescence under light-adapted conditions. QY was calculated as (Fm′ − F_t_)/Fm′, where Fm′ represents the maximal fluorescence under light-adapted conditions.

### 2.8. Data Processing

The data were analyzed using univariate analysis of variance (ANOVA) and Duncan’s multiple comparison test. Statistical significance was established at a 5% level (*p* < 0.05). SPSS 19.0 statistical software (SPSS Inc., Chicago, IL, USA) was utilized for data analysis, and diagrams were plotted with GraphPad Prism 9.0 (San Diego, CA, USA).

## 3. Results

### 3.1. Concentration-Dependent Effects of PLA on the Growth and Biomass Accumulation of FACHB-82

To evaluate the effect of PLA on the growth of FACHB-82, a standard linear fitting curve between algal cell concentration and absorbance value (OD_680_) was first established (Figure S2). The relationship between algal biomass and OD_680_ values of the algae was described using the regression equation Y = 295.39X + 3.3333, where X and Y are the absorbance value and algal biomass, respectively. A strong linear relationship was also observed with a coefficient of determination (R^2^) value of 0.9938, and this relationship was subsequently used to estimate biomass from OD_680_ measurements.

**Figure 1:**
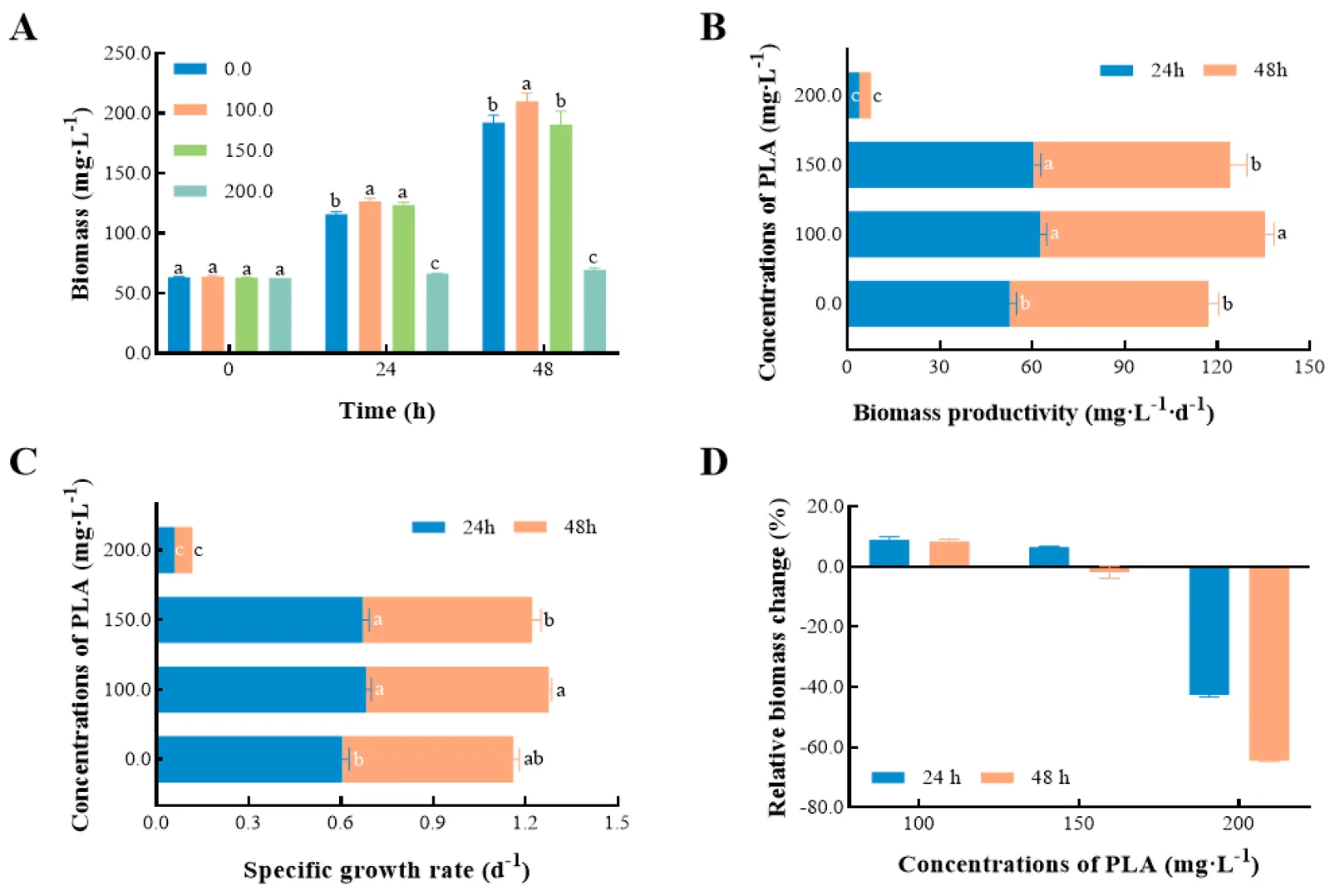
Effects of PLA on the growth of FACHB-82. (**A**) Biomass accumulation of FACHB-82 after exposure to different concentrations of PLA for 0, 24 and 48 h. (**B**) Biomass productivity under different PLA treatments at 24 and 48 h. (**C**) Specific growth rate under different PLA treatments at 24 and 48 h. (**D**) Relative biomass change after 24 and 48 h of PLA exposure compared with the untreated cells. Positive values indicate increased biomass relative to the control, whereas negative values indicate decreased biomass relative to the control. Values represent the mean of three independent measurements, and bars indicate SD. Different letters indicate a significant difference at the 0.05 level (*p* < 0.05, Duncan’s multiple range test).

FACHB-82 was cultured in different PLA concentrations to study the effect of PLA on the algal biomass accumulation. As shown in Figure 1A, PLA exposure affected the biomass accumulation of FACHB-82 in a concentration-dependent manner. After 24 h of exposure, the biomass in the control group was 116.07 mg·L^−1^, and treatments with 100.0 and 150.0 mg·L^−1^ PLA increased the biomass to 126.51 mg·L^−1^ and 123.66 mg·L^−1^, respectively. In contrast, exposure to 200 mg·L^−1^ PLA markedly reduced the biomass to 66.55 mg·L^−1^. After 48 h, the 100.0 mg·L^−1^ PLA treatment resulted in the highest biomass (210.01 mg·L^−1^), while the 150.0 mg·L^−1^ treatment showed a biomass level comparable to that of the control. As expected, biomass remained significantly suppressed under 200.0 mg·L^−1^ PLA treatment, with a value of 69.70 mg·L^−1^. The above results indicate that low concentrations of PLA did not markedly inhibit algal growth compared with the control. In particular, treatments with 100.0 and 150.0 mg·L^−1^ PLA resulted in slightly increased biomass accumulation, suggesting that these concentrations may induce a mild growth-promoting or adaptive response in FACHB-82 (Figure 1A). However, the biomass of FACHB-82 under 200.0 mg·L^−1^ PLA treatment was substantially lower than that of the control and the low-concentration treatment groups, and this inhibitory effect became more pronounced with prolonged exposure.

Consistent with biomass accumulation, biomass productivity was also strongly affected by PLA exposure (Figure 1B). At 24 h, the 100.0 and 150.0 mg·L^−1^ PLA treatments showed higher biomass productivity (62.52 and 60.46 mg·L^−1^·d^−1^) than that of the control group (52.68 mg·L^−1^·d^−1^). However, biomass productivity significantly decreased to 3.8 mg·L^−1^·d^−1^ under 200.0 mg·L^−1^ PLA treatment. At 48 h, the highest productivity was observed in the 100 mg·L^−1^ PLA treatment, reaching 73.01 mg·L^−1^·d^−1^, followed by the 150.0 mg·L^−1^ treatment and the control group. Biomass productivity under the 200.0 mg·L^−1^ PLA treatment remained the lowest throughout the exposure period. Furthermore, a similar trend was observed for the specific growth rate. The 100.0 mg·L^−1^ PLA treatment maintained the highest specific growth rate, which was 12.73% and 13.12% higher than that of the control group at 24 h and 48 h. However, the 200 mg·L^−1^ exposure severely inhibited the algal specific growth rate (Figure 1C).

The relative biomass change further confirmed the biphasic effect of PLA on the growth of FACHB-82 (Figure 1D). Compared with the control, 100.0 mg·L^−1^ PLA treatment increased biomass by approximately 8.99% and 8.35% at both 24 and 48 h. The 150.0 mg·L^−1^ PLA treatment increased biomass by 6.53% at 24 h but showed a slight decrease of 1.86% at 48 h. Meanwhile, 200.0 mg·L^−1^ PLA significantly reduced biomass by 42.66% and 64.38% at 24 and 48 h, respectively. These results demonstrate that PLA exerted concentration-dependent and time-dependent effects on algal growth, with low PLA concentrations slightly promoting biomass accumulation and high PLA concentrations strongly suppressing algal growth.

### 3.2. PLA Modulates Photosynthetic Pigment Synthesis in FACHB-82

**Figure 2:**
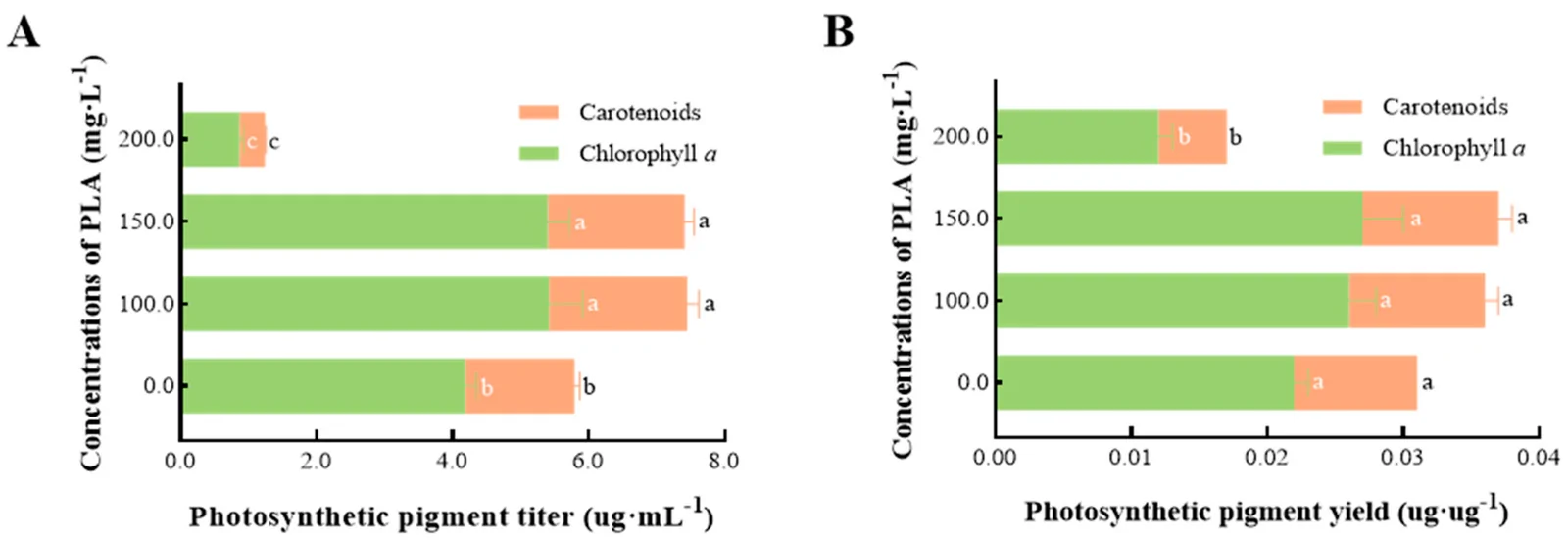
Effects of PLA on photosynthetic pigment accumulation in FACHB-82 after 48 h of exposure. (**A**) Photosynthetic pigment titers in FACHB-82 after exposure to different concentrations of PLA. (**B**) Biomass-normalized photosynthetic pigment yields under different PLA treatments. Values represent the mean of three independent measurements, and bars indicate SD. Different letters indicate a significant difference at the 0.05 level (*p* < 0.05, Duncan’s multiple range test).

To determine whether the growth response of FACHB-82 to PLA was accompanied by changes in photosynthetic pigment accumulation, we quantified the titers of chlorophyll *a* and carotenoids after PLA exposure. As shown in Figure 2A, PLA altered pigment accumulation in a concentration-dependent manner. Compared with the control, 100.0 and 150.0 mg·L^−1^ PLA increased the titer of total pigment, mainly due to higher chlorophyll *a* synthesis. Specifically, the titer of chlorophyll *a* increased from 4.184 μg·mL^−1^ in the control to 5.422 and 5.401 μg·mL^−1^ under 100.0 and 150.0 mg·L^−1^ PLA, respectively. Meanwhile, the titers of carotenoid showed a smaller increase, reaching 2.028 and 2.008 μg·mL^−1^ under 100.0 and 150.0 mg·L^−1^ PLA, respectively. However, 200.0 mg·L^−1^ PLA markedly reduced both chlorophyll *a* and carotenoid titers, indicating impaired pigment accumulation at the higher PLA concentration.

We also examined the effect of different PLA concentrations on the photosynthetic pigment yield (normalized to biomass) of algal cells, and algal photosynthetic pigment yield showed a distinct trend compared to pigment titer (Figure 2B). Although 100.0 and 150.0 mg·L^−1^ PLA increased pigment titers, their pigment yields were only slightly changed relative to the control. The highest pigment yield was observed under 150.0 mg·L^−1^ PLA, whereas the 100.0 mg·L^−1^ PLA treatment showed a pigment yield comparable to that of the control. Notably, 200.0 mg·L^−1^ PLA significantly decreased pigment yield despite the marked reduction in biomass and pigment titer, suggesting that the high PLA exposure suppressed pigment accumulation together with cell growth rather than simply diluting pigment content through biomass changes.

### 3.3. PLA Perturbs PSII Photochemical Performance in FACHB-82

**Figure 3:**
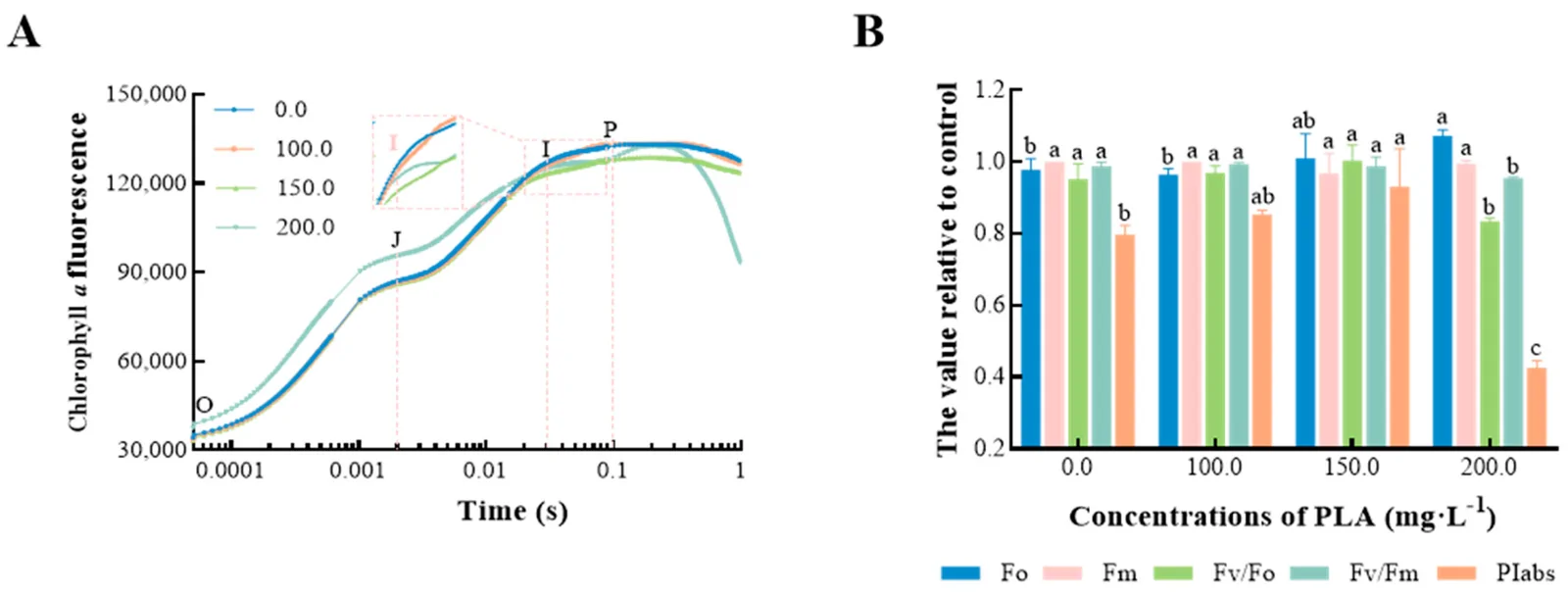
Effects of PLA on chlorophyll *a* fluorescence transients in FACHB-82 after 48 h of exposure. (**A**) Fast chlorophyll *a* fluorescence induction curves, known as OJIP transients, in FACHB-82 exposed to different concentrations of PLA. The O, J, I and P steps are indicated. The inset shows an enlarged view of the O–J phase. (**B**) Relative changes in OJIP-derived parameters under different PLA treatments, including initial fluorescence (Fo), maximal fluorescence (Fm), variable-to-initial fluorescence ratio (Fv/Fo), maximum quantum efficiency of PSII (Fv/Fm), and performance index on absorption basis (PIabs). All values were normalized to the untreated control. Values represent the mean of three independent measurements, and bars indicate SD. Different letters indicate a significant difference at the 0.05 level (*p* < 0.05, Duncan’s multiple range test).

To determine whether PLA affected the photosynthetic apparatus of FACHB-82, chlorophyll *a* fluorescence induction transients (OJIP) were recorded, and OJIP-derived parameters were analyzed. The OJIP curves exhibited typical O–J–I–P phases in all treatments, indicating that PSII remained functionally detectable under PLA exposure (Figure 3A). However, the shape and amplitude of the curves were altered by PLA. The curves of the control group and the 100.0 mg·L^−1^ and 150.0 mg·L^−1^ PLA treatment groups were broadly comparable, whereas the 200 mg·L^−1^ PLA exposure increased the level of J step, but strongly decreased the I- and P-step levels, suggesting impaired stability of PSII electron transport under high PLA exposure.

Quantitative analysis of fluorescence parameters further supported this viewpoint (Figure 3B). Low PLA concentrations caused slight changes in Fo, Fm, Fv/Fo and Fv/Fm values relative to the control. In particular, 100.0 and 150.0 mg·L^−1^ PLA maintained Fv/Fm at levels close to the control, indicating that the maximum quantum efficiency of PSII was largely preserved under these treatments. However, 200.0 mg·L^−1^ PLA significantly altered the values of the above fluorescence parameters. For example, the Fo value increased slightly, whereas the Fm value remained unchanged, resulting in reduced Fv/Fo and Fv/Fm values. These changes indicate that high PLA concentration impaired PSII photochemical efficiency. Furthermore, the performance index on an absorption basis, PIabs, showed the greatest sensitivity to PLA exposure. Compared with the control, PIabs was slightly increased under 100.0 and 150.0 mg·L^−1^ PLA; however, a significant downward trend was observed at a concentration of 200 mg·L^−1^. It is worth noting that the decrease in PIabs is more significant than that in Fv/Fm, indicating that PIabs is more sensitive to capturing early changes in PSII performance. These results indicate that high PLA concentration impaired PSII functionality, while low PLA concentrations exerted only minor effects on PSII primary photochemistry.

### 3.4. PLA Accelerates the OJIP Rise and Alters PSII Electron Transport Dynamics in FACHB-82

**Figure 4:**
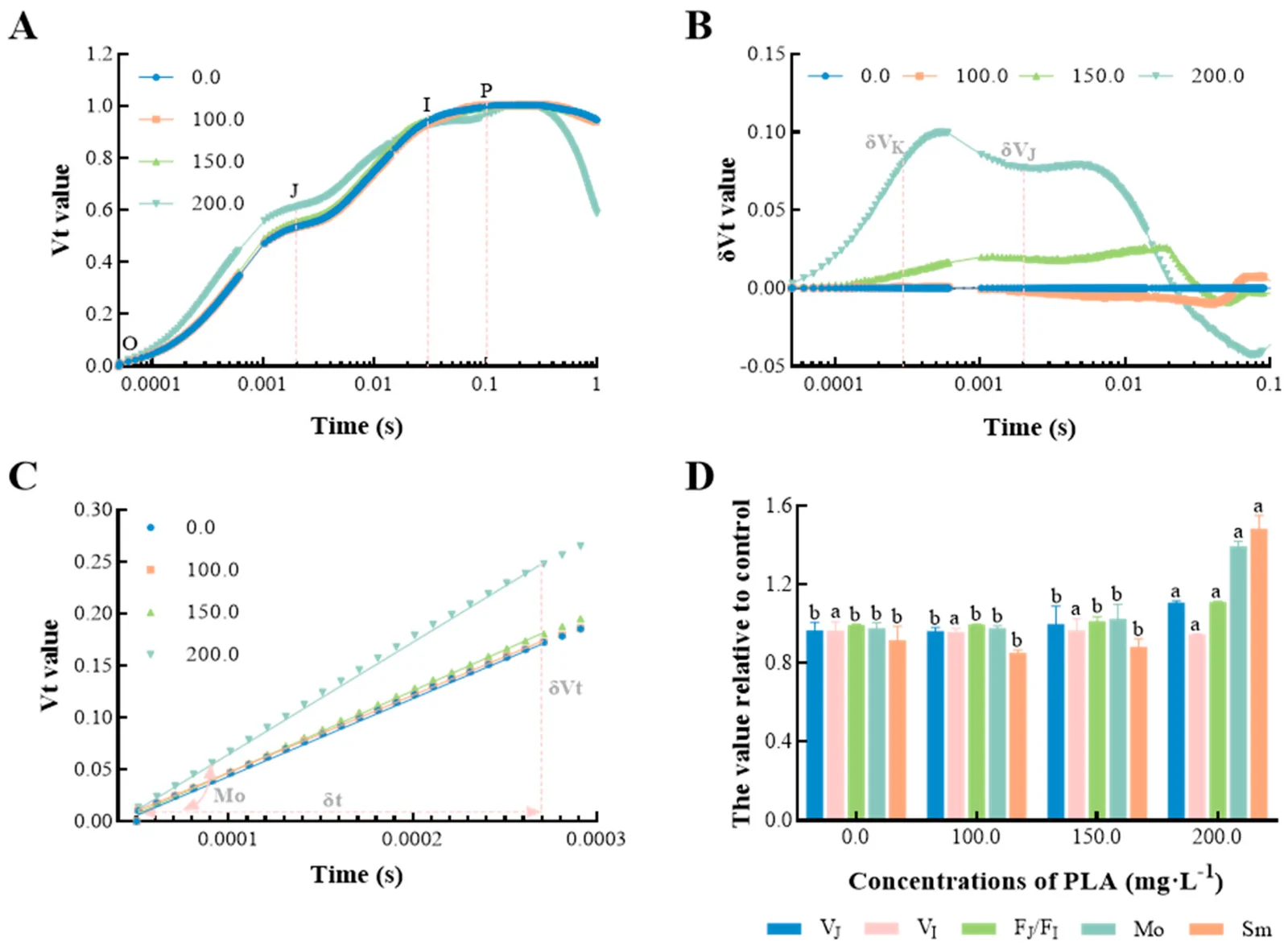
Effects of PLA on relative variable fluorescence and OJIP-derived parameters in FACHB-82 after 48 h of exposure. (**A**) Relative variable fluorescence curves normalized between Fo and Fm as Vt = (Ft − Fo)/(Fm − Fo). (**B**) Difference kinetics calculated as δVt = Vt_(treatment)_ − Vt_(control)_. δVt indicate the difference at the J step. (**C**) Initial rise of Vt curves from 50 to 300 μs plotted on a linear time scale for estimating Mo. (**D**) Relative values of V_J_, V_I_, F_J_/F_I_, Mo and Sm under different PLA treatments. Difference kinetics in (**B**) were calculated by subtracting the mean normalized control curve from the corresponding mean normalized treatment curve and are presented for descriptive purposes only. For (**D**), values represent the mean of three independent measurements, and bars indicate SD. Different letters indicate a significant difference at the 0.05 level (*p* < 0.05, Duncan’s multiple range test).

To reveal subtle changes in the OJIP fluorescence transient, the fluorescence curves were double-normalized between Fo and Fm and expressed as relative variable fluorescence (Vt). As shown in Figure 4A, all treatments exhibited the typical O–J–I–P rise, but PLA altered the trajectory of the normalized fluorescence transient. Compared with the control, 100.0 and 150.0 mg·L^−1^ PLA produced only modest changes in Vt, whereas 200.0 mg·L^−1^ PLA caused a faster rise during the O–J phase and a higher relative fluorescence level around the J step, indicating that high PLA exposure modified the reduction kinetics of the PSII electron transport chain. The difference kinetics, calculated as δVt = Vt(treatment) − Vt(control), further separated the effects of PLA on distinct phases of the OJIP transient (Figure 4B). The 200.0 mg·L^−1^ treatment generated the largest positive δVt signal during the O–J phase, with a clear maximum signal around the J step, indicating enhanced accumulation of reduced Q_A_ or restricted electron flow beyond Q_A_. In contrast, the 100.0 mg·L^−1^ treatment showed only minor deviations from the control, while 150.0 mg·L^−1^ PLA induced a weak but detectable increase in δVt. These results suggest that low PLA concentration had limited effects on the early fluorescence rise, whereas high PLA concentration may result in the inactivation of PSII reaction centers.

The initial part of the Vt curve was then plotted on a linear time scale to evaluate the initial slope of fluorescence rise (Mo). The slope was markedly steeper under 200.0 mg·L^−1^ PLA than that in the control and lower PLA treatments, indicating a faster closure of PSII reaction centers (Figure 4C). As expected, quantitative OJIP parameters showed that the Mo value under 200.0 mg·L^−1^ PLA treatment was 42.72% higher that that under control (Figure 4D). For further investigation into the initial action site of PLA, a set of JIP-test parameters were recorded, and V_J_, Sm, and F_J_/F_I_ presented a rising tendency, with V_I_ being the only exception. The increase in V_J_ supports a stronger limitation of electron transfer from Q_A_ to downstream electron acceptors, while the rise in Sm suggests that high PLA exposure altered the size or turnover requirement of the electron acceptor pool. These results indicate that high PLA exposure accelerated PSII reaction-center closure and promoted Q_A_ reduction, while low to moderate PLA concentrations had only minor effects on PSII electron transport dynamics.

### 3.5. High PLA Exposure Induces a Positive L-Band in FACHB-82

**Figure 5:**
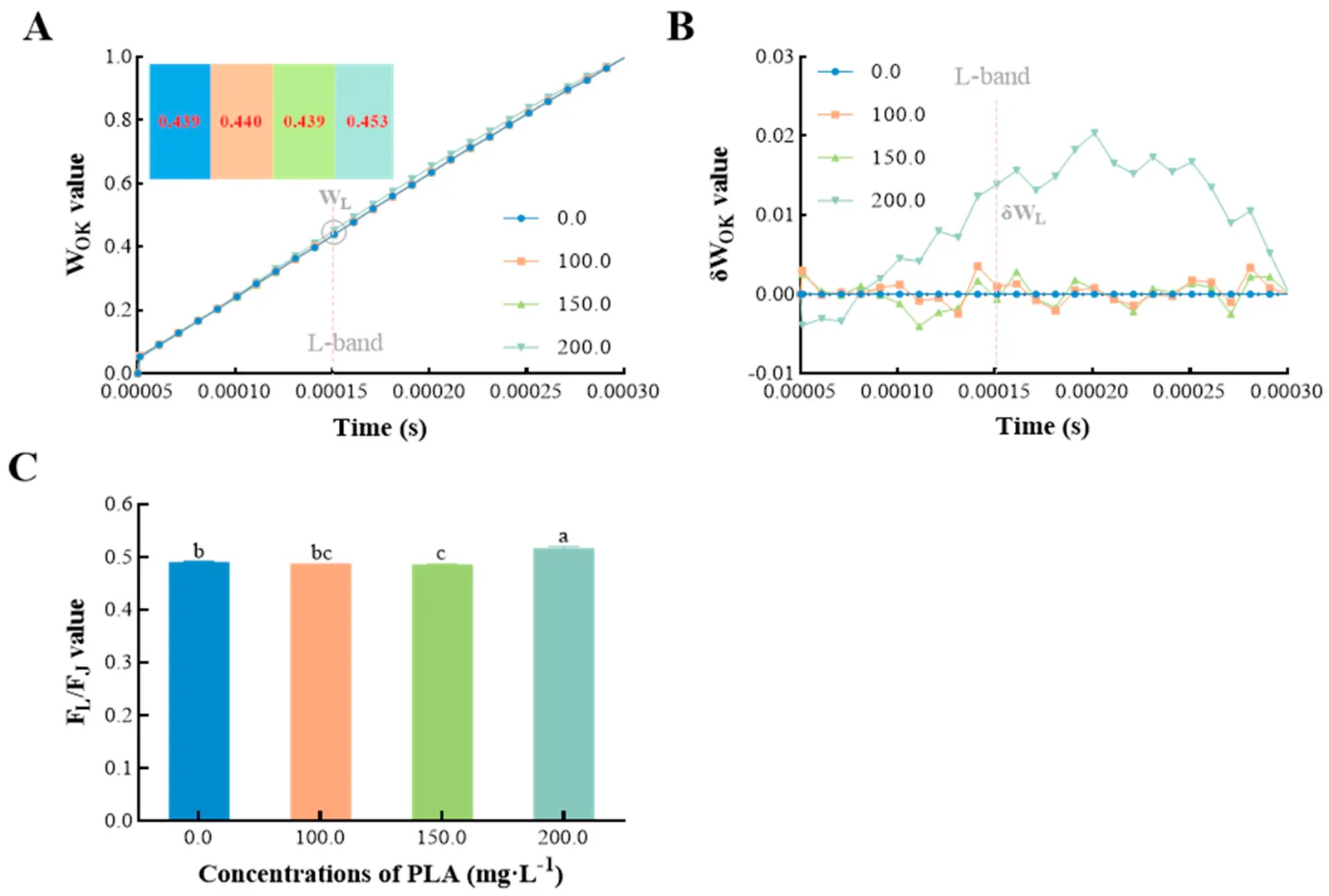
Effects of PLA on the L-band of chlorophyll fluorescence transients in FACHB-82 after 48 h of exposure. (**A**) Fluorescence rise kinetics double-normalized between the O step at 50 μs and the K step at 300 μs as W_OK_ = (F_t_ − Fo)/(F_K_ − Fo). (**B**) Difference kinetics calculated as δW_OK_ = W_OK(treatment)_ − W_OK(control)_, showing the L-band region. (**C**) F_L_/F_J_ values under different PLA treatments. Difference kinetics in (**B**) were calculated by subtracting the mean normalized control curve from the corresponding mean normalized treatment curve and are presented for descriptive purposes only. For (**C**), values represent the mean of three independent measurements, and bars indicate SD. Different letters indicate a significant difference at the 0.05 level (*p* < 0.05, Duncan’s multiple range test).

To examine whether PLA affected the energetic connectivity among PSII units, the O–K phase of the chlorophyll fluorescence transient was further investigated (Figure 5). The fluorescence rise kinetics were double-normalized between the O step at 50 μs and the K step at 300 μs and expressed as W_OK_ = (Ft − Fo)/(F_K_ − Fo). As shown in Figure 5A, the normalization kinetics revealed insignificant differences among the control, 100.0 and 150.0 mg·L^−1^ PLA treatments, whose W_OK_ curves almost overlapped throughout the O–K phase. Meanwhile, the 200.0 mg·L^−1^ PLA treatment showed a slight but consistent elevation in the O–K phase. Subsequently, the difference kinetics, calculated as δW_OK_ = W_OK_(treatment) − W_OK_(control), further demonstrated the above response (Figure 5B). The δW_OK_ values under 100.0 and 150.0 mg·L^−1^ PLA treatments were close to those of the control, suggesting that low concentrations of PLA exerted negligible effects on the O–K transition. By contrast, the 200.0 mg·L^−1^ PLA treatment induced an obvious positive L-band, with significant differences observed in the L-band phase, indicating that high concentrations of PLA disrupted the coordination between antenna excitation and PSII reaction-center trapping. Furthermore, quantitative analysis of the F_L_/F_J_ ratio supported the L-band response. It was observed that F_L_/F_J_ remained relatively stable under 100.0 and 150.0 mg·L^−1^ PLA, whereas 200.0 mg·L^−1^ PLA significantly increased F_L_/F_J_ relative to the control and the lower-concentration treatments (Figure 5C). Specifically, under 200.0 mg·L^−1^ PLA treatment, the F_L_/F_J_ value increased from 0.491 in the control to 0.517, confirming a significant enhancement of the L-band signal. These results indicate that high PLA concentration perturbed the energy transfer architecture of PSII, whereas low PLA concentrations had minor effects.

### 3.6. High PLA Exposure Induces a Positive K-Band and Impairs the Donor Side of PSII in FACHB-82

**Figure 6:**
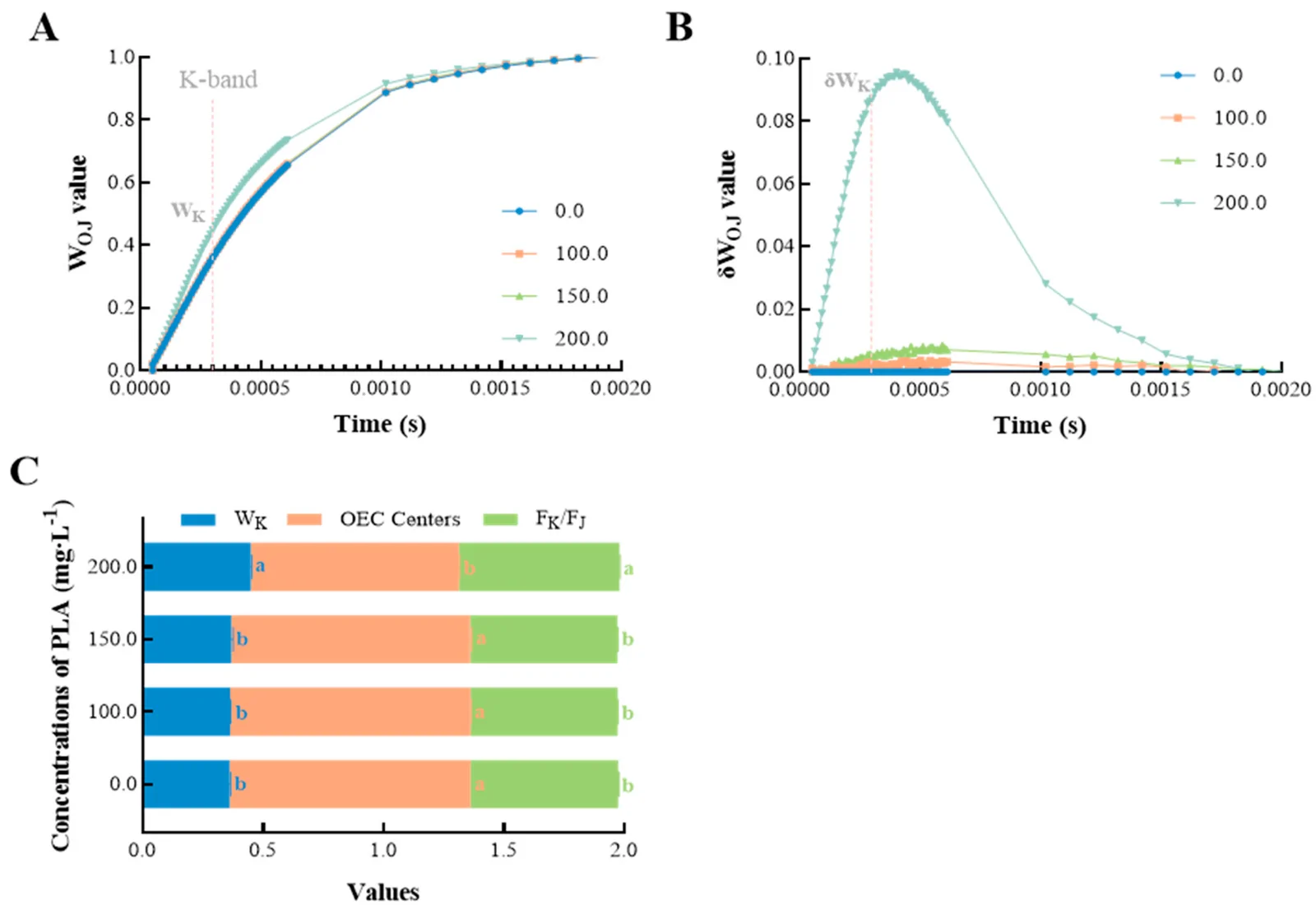
Effects of PLA on the K-band of chlorophyll fluorescence transients in FACHB-82 after 48 h of exposure. (**A**) Fluorescence rise kinetics normalized between the O step (50 μs) and the J step (2 ms), expressed as W_OJ_ = (F_t_ − Fo)/(F_J_ − Fo). (**B**) Difference kinetics calculated as δW_OJ_ = W_OJ(treatment)_ − W_OJ(control)_, showing the K-band region. (**C**) Relative values of W_K_, OEC centers and F_K_/F_J_ under different PLA treatments. Difference kinetics in (**B**) were calculated by subtracting the mean normalized control curve from the corresponding mean normalized treatment curve and are presented for descriptive purposes only. For (**C**), values represent the mean of three independent measurements, and bars indicate SD. Different letters indicate a significant difference at the 0.05 level (*p* < 0.05, Duncan’s multiple range test).

To assess the effects of PLA on the donor-side function of PSII, chlorophyll fluorescence transient curves were double-normalized between the O step at 50 μs and the J step at 2 ms, and denoted as W_OJ_. As shown in Figure 6, the W_OJ_ curves of the control and the 100.0 mg·L^−1^ and 150.0 mg·L^−1^ PLA treatments were largely overlapped, demonstrating that low PLA concentrations exerted negligible influences on the photochemical processes at the PSII donor side (Figure 6A). In comparison, the 200.0 mg·L^−1^ PLA treatment induced a marked upward deviation in the O–K phase, implying that high PLA concentration altered the kinetic characteristics of the fluorescence rise. Then, this trend became even more evident after subtracting the control curve. The difference kinetics, expressed as δW_OJ_ = W_OJ_(treatment) − W_OJ_(control), showed that the 100.0 and 150.0 mg·L^−1^ treatments caused only minor deviations from control, whereas 200.0 mg·L^−1^ PLA induced a pronounced positive K-band with a marked peak in the K-step phase (Figure 6B). Parameter-based analysis further corroborated the above kinetic trends. Compared with the control, 200.0 mg·L^−1^ PLA significantly increased W_K_ and F_K_/F_J_, while decreasing the relative abundance of OEC centers (Figure 6C). In contrast, 100.0 and 150.0 mg·L^−1^ PLA exerted negligible or insignificant change on these parameters, demonstrating that high PLA concentrations impair the water-splitting capacity and diminish electron supply to PSII. Since a positive K-band is widely recognized as a typical indicator of impaired electron donation from the oxygen-evolving complex to PSII reaction centers, these findings indicate that high PLA exposure substantially disrupts the donor-side function of PSII.

### 3.7. PLA Alters the I–P Phase and Terminal Electron Acceptor Dynamics in FACHB-82

**Figure 7:**
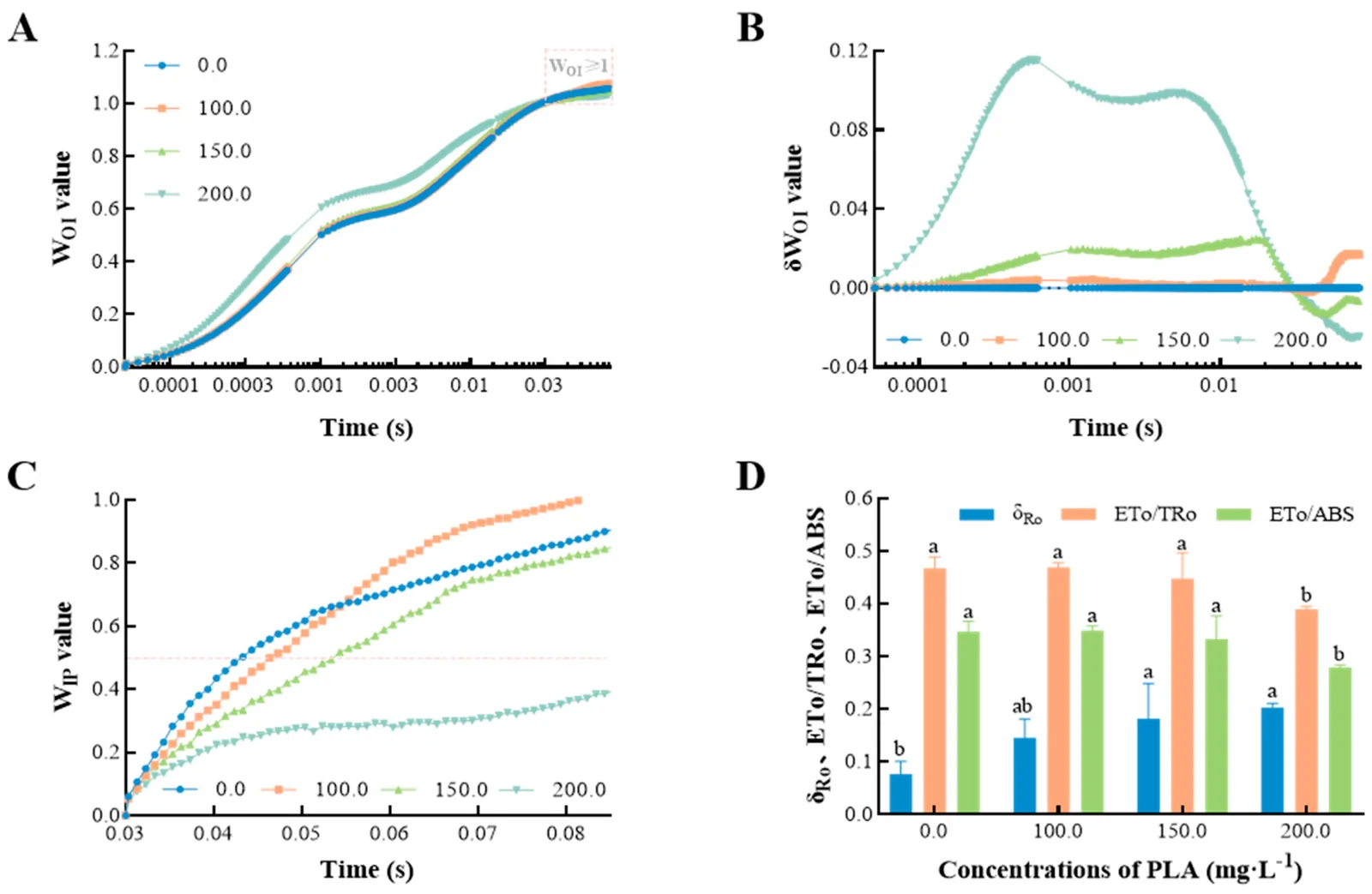
Effects of PLA on the I–P phase and electron transport efficiency in FACHB-82 after 48 h of exposure. (**A**) Fluorescence rise kinetics normalized between the O step and I step as W_OI_ = (Ft − Fo)/(F_I_ − Fo). (**B**) Difference kinetics calculated as δW_OI_ = W_OI(treatment)_ − W_OI(control)_. (**C**) Fluorescence rise kinetics normalized between the I step and P step as W_IP_ = (F_t_ − F_I_)/(F_P_ − F_I_). The dashed line indicates W_IP_ = 0.5. (**D**) Relative values of δ_Ro_, ETo/TRo and ETo/ABS under different PLA treatments. Difference kinetics in (**B**) were calculated by subtracting the mean normalized control curve from the corresponding mean normalized treatment curve and are presented for descriptive purposes only. For (**D**), values represent the mean of three independent measurements, and bars indicate SD. Different letters indicate a significant difference at the 0.05 level (*p* < 0.05, Duncan’s multiple range test).

To evaluate the effect of PLA on the I–P phase of the OJIP transient, fluorescence curves were normalized between the O step and I step on a logarithmic time scale and denoted as W_OI_. The W_OI_ ≥ 1 segment was plotted over the linear time range of 30–85 ms to characterize the I–P phase. All treatments maintained a clear O–J–I transient transition, whereas the W_OI_ kinetics differed substantially across PLA concentrations (Figure 7A). The 100.0 and 150.0 mg·L^−1^ PLA treatment exhibited a W_OI_ trend comparable to or slightly higher than that of the control during the I–P phase. In contrast, 200.0 mg·L^−1^ PLA induced a notable elevation during the O–I phase, followed by a distinctly restricted increase in the W_OI_ ≥ 1 phase. The enlarged view of W_OI_ ≥ 1 further demonstrates the effects of PLA on the I–P phase, and different PLA concentrations reduced the amplitude of W_OI_ curves to varying extents (Figure S3). The 100.0 mg·L^−1^ group exhibited the greatest rise in W_OI_ during this interval, while the 150.0 mg·L^−1^ group maintained a lower W_OI_ level than the control throughout the I–P phase. In comparison, the 200.0 mg·L^−1^ treatment had the minimum W_OI_ amplitude, indicating that high PLA exposure markedly suppresses the amplitude of the I–P phase. Then, δW_OI_ was used to show the effects of PLA on the J step, and the results were in accordance with that of δVt (Figure 7B).

The I–P phase was further analyzed using W_IP_, which was normalized between the I step and P step. As shown in Figure 7C, the control and 100.0 mg·L^−1^ treatment presented a gradual increase in W_IP_ and surpassed the half rise-time value (W_IP_ = 0.5) during the observation period, suggesting a relatively fast progression of the I–P phase. Meanwhile, the 150.0 mg·L^−1^ treatment had a slower W_IP_ elevation, whereas the 200.0 mg·L^−1^ treatment stayed below the 0.5 level for the entire duration of the analysis, demonstrating that PLA at 200.0 mg·L^−1^ significantly delayed or inhibited the reduction rates of the end electron acceptors on PSI. Parameter-based analysis further showed that 200.0 mg·L^−1^ PLA increased δRo but decreased ETo/TRo and ETo/ABS, indicating that high PLA exposure altered downstream electron-transfer dynamics and reduced overall electron-transport efficiency (Figure 7D).

### 3.8. PLA Reshapes PSII Energy Allocation and Non-Photochemical Quenching in FACHB-82

PSII energy flux parameters were analyzed to further assess how PLA affected energy allocation in FACHB-82. As shown in Figure S4, energy flux analysis showed that 200.0 mg·L^−1^ PLA increased ABS/RC, TRo/RC and DIo/RC, whereas ETo/RC showed comparatively smaller changes. Meanwhile, low PLA concentrations caused limited changes in these parameters, thus indicating that high PLA exposure altered the balance between energy absorption, trapping and dissipation at the PSII reaction center.

**Figure 8:**
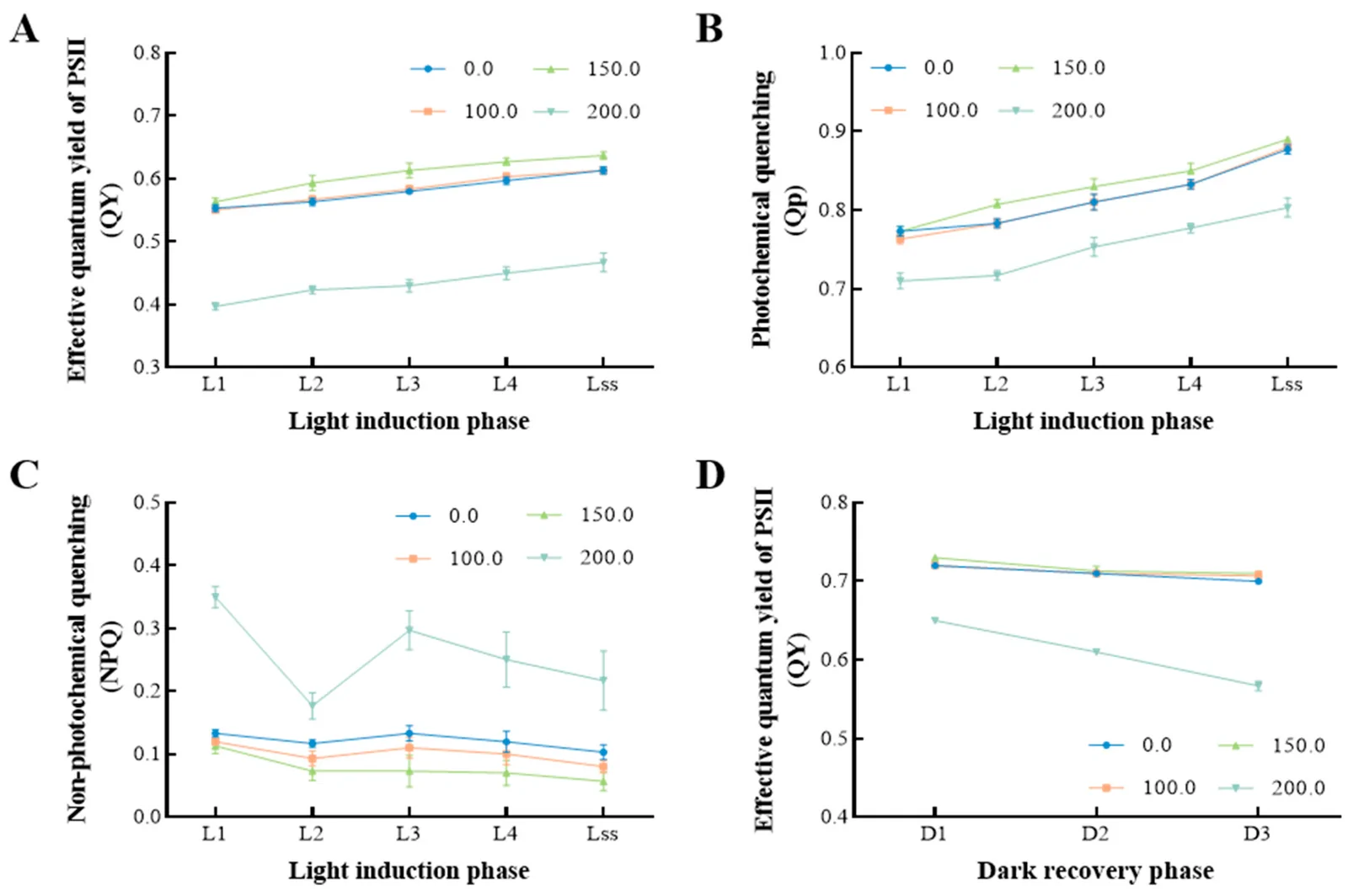
Effects of PLA on PSII photochemical performance and non-photochemical quenching in FACHB-82 after 48 h of exposure. (**A**) Effective quantum yield of PSII (QY) during the light induction phase. (**B**) Photochemical quenching (Qp) during the light induction phase. (**C**) Non-photochemical quenching (NPQ) during the light induction phase. (**D**) Effective quantum yield (QY) of PSII during the dark recovery phase. L1, L2, L3, L4 and Lss correspond to sequential light-adapted measurement points during the 60 s actinic illumination phase, with saturating pulses applied at approximately 7, 19, 31, 43 and 55 s, respectively. D1, D2 and D3 represent sequential dark-recovery measurement points after switching off the actinic light. These parameters were used to evaluate the recovery capacity of PSII after light exposure. Values represent the mean of three independent measurements, and bars indicate SD.

Subsequently, NPQ-related fluorescence kinetics were analyzed to reveal quenching regulation. During the light induction phase, the effective quantum yield (QY) of PSII increased gradually from L1 to Lss in all treatments, but the magnitude of QY differed among PLA concentrations (Figure 8A). The control and the 100.0 and 150.0 mg·L^−1^ treatments maintained relatively high QY values throughout the induction phase, and 200.0 mg·L^−1^ PLA showed the lowest QY from L1 to Lss. At the steady-state stage, QY under 200.0 mg·L^−1^ PLA was 23.82% lower than that in the control, demonstrating that high PLA exposure reduced the effective photochemical efficiency of PSII.

Also, a similar trend was observed for photochemical quenching (qP). As shown in Figure 8B, qP followed an increase trend during the light induction phase among all groups, indicating a gradual adjustment of PSII reaction center openness. However, qP values under 200.0 mg·L^−1^ PLA remained consistently lower than those in the control and the low-concentration treatments, showing that high PLA concentration will reduce the photochemical quenching during light adaptation. However, non-photochemical quenching displayed a distinct response trend. NPQ was strongly enhanced by 200.0 mg·L^−1^ PLA at all light-adapted stages, whereas NPQ remained low and relatively stable in the control and the 100.0 and 150.0 mg·L^−1^ treatments (Figure 8C), indicating that high-concentration PLA exposure induced a stronger non-photochemical quenching response during light adaptation.

Finally, dark recovery analysis was used to evaluate the short-term recovery of PSII photochemical performance after actinic illumination. As shown in Figure 8D, during dark recovery, QY was largely maintained in the control and low-concentration PLA treatments, but gradually declined under 200.0 mg·L^−1^ PLA from D1 to D3. At the D3 stage, the QY value at 200.0 mg·L^−1^ PLA reached 0.567, which was 19.00% lower than that in the control. The above results indicate that high PLA concentration reduced photochemical efficiency and the openness of the reaction center, while enhancing non-photochemical quenching, accompanied by limited short-term recovery of PSII quantum yield after actinic illumination, indicating a strong shift from photochemical use to energy dissipation under 200.0 mg·L^−1^ PLA exposure.

## 4. Discussion

### 4.1. Biphasic Growth Response of FACHB-82 to PLA

The frequent occurrence of cyanobacterial blooms is closely associated with the increasing eutrophication of water bodies, largely driven by nutrient enrichment from agricultural runoff, domestic wastewater and industrial discharges [2,31,32]. Such eutrophication-driven cyanobacterial blooms pose a persistent challenge to freshwater restoration and water quality management, because nutrient enrichment promotes harmful algal blooms and contributes to water-quality deterioration and loss of aquatic ecosystems [33]. Filamentous cyanobacteria are important contributors to freshwater cyanobacterial blooms and often play key roles in bloom formation, persistence and nitrogen cycling [34]. At present, naturally derived allelochemicals have gained attention as eco-friendly agents for suppressing bloom-forming cyanobacteria, and their inhibitory effects on cyanobacteria have been shown to depend on compound structure, exposure concentration and target species [14,16,17,35]. PLA has garnered significant research interest owing to its broad-spectrum antibacterial and antifungal properties [19,20]; however, the inhibitory effects of PLA on the growth of filamentous cyanobacteria and its underlying photosynthetic mechanism remain poorly understood. FACHB-82 is a representative filamentous cyanobacterium frequently associated with freshwater blooms, and its growth depends strongly on the coordinated function of photosynthetic energy conversion and electron transport. Consequently, evaluating the response of FACHB-82 to PLA can provide useful insight into the anticyanobacterial potential of PLA and help clarify whether PSII is involved in PLA-induced inhibition.

In our preliminary study, 100.0 mg·L^−1^ PLA slightly enhanced algal biomass accumulation, biomass productivity and specific growth rate, while 150.0 mg·L^−1^ produced a weaker growth-maintaining effect. In contrast, 200.0 mg·L^−1^ PLA strongly suppressed algal biomass accumulation, and this inhibition became more pronounced with longer exposure. This biphasic effect is consistent with that of other allelochemicals, whose biological effects may shift from stimulation or acclimation at low-concentration treatment to inhibition under higher-concentration exposure [16,17]. However, only a limited concentration range was examined, and the present study does not establish an optimal PLA concentration for controlling FACHB-82. Furthermore, this biphasic response was also reflected in photosynthetic pigment accumulation. The increase in chlorophyll *a* and carotenoid titers under 100.0 and 150.0 mg·L^−1^ PLA suggests that low concentrations of PLA exposure maintained or even promoted pigment accumulation, which may partly explain the sustained growth observed under these treatments. In contrast, the marked decrease in pigment titers under 200.0 mg·L^−1^ PLA was consistent with the significant reduction in biomass, indicating that high PLA concentration impaired both cell growth and photosynthetic pigment accumulation. Previous studies showed that phenolic acids, such as ferulic acid and *p*-hydroxybenzoic acid, inhibited algal growth and chlorophyll *a* content of *Anabaena flos-aquae* [36]. Also, Li et al. (2024) reported that the photosynthetic pigment content of *M. aeruginosa* was significantly inhibited in algal cells after exposure to the supernatant of *L. mesenteroides* DH strain, in which PLA was one of the active substances [23]. These findings were consistent with our results. Consequently, the growth-inhibitory effect of high PLA exposure was closely associated with a decline in the photosynthetic capacity of FACHB-82.

### 4.2. OJIP Analysis Reveals That High PLA Exposure Impairs PSII Primary Photochemistry

Chlorophyll *a* fluorescence rise kinetics (OJIP transient), which have been used as a sensitive, rapid and non-invasive approach for probing stress-induced changes in the photosynthetic apparatus, record the polyphasic rise in chlorophyll fluorescence from the initial O step to the maximal P step, reflecting the stagewise reduction of electron carriers in PSII and the downstream photosynthetic electron transport chain [37,38]. JIP-test parameters translate the OJIP fluorescence transient into quantitative information on PSII structure and function, including reaction-center activity, excitation energy trapping, electron transport and energy dissipation [39], and have been widely used to assess stress-induced changes in photosynthetic performance, because different phases of the OJIP transient provide information on distinct segments of the photosynthetic electron transport chain [40].

In this study, three different concentrations of PLA were used to measure and analyze the characteristics of the OJIP transient of FACHB-82. Low PLA concentrations caused only minor changes in the OJIP curves, suggesting that PSII primary photochemical processes were largely maintained under these treatments. By contrast, 200.0 mg·L^−1^ PLA markedly altered the fluorescence rise kinetics, particularly during the O–J phase, indicating that the early stage of PSII electron transport was strongly affected. Furthermore, several fluorescence parameters also provide data support for the above changes. Fv/Fm mainly reflects the maximum quantum efficiency of PSII primary photochemistry, whereas PIabs provides a more integrative assessment of PSII performance by combining reaction-center density, excitation energy trapping and electron transport beyond Q_A_^−^ [26]. In the present study, Fv/Fm and PIabs decreased under 200.0 mg·L^−1^ PLA treatment, and the stronger decrease in PIabs indicates that high PLA exposure impaired the integrated functional state of PSII rather than causing only a limited reduction in maximum photochemical efficiency. Previous studies have demonstrated that aromatic organic acids can interfere with cyanobacterial photosynthetic activity. For instance, Liu et al. reported that water dropwort-derived aromatic acids, including benzoic acid, salicylic acid and ferulic acid, contributed to the growth inhibition of *M. aeruginosa* and affected photosynthetic performance [17], which was consistent with our research findings.

Subsequently, the fluorescence curves double-normalized between Fo and Fm were presented as relative variable fluorescence (Vt transient), and an increase in Vt transient around the J step generally reflects enhanced accumulation of reduced Q_A_^−^ because of the blocking of the electron flow beyond Q_A_. In our study, 200.0 mg·L^−1^ PLA increased Vt and produced a positive δVt around the J step, indicating pronounced perturbation of electron transport beyond Q_A_. Meanwhile, V_J_ indicates the relative variable fluorescence at the J step and is commonly used to estimate the extent of PSII reaction-center closure at this phase, whereas Mo, the approximated initial slope of the normalized fluorescence rise, reflects the initial rate of PSII reaction-center closure. The increased V_J_ and Mo observed in our study are consistent with faster reaction-center closure and greater Q_A_^−^ accumulation in FACHB-82. Furthermore, the increases in V_J_ and F_J_/F_I_ under 200.0 mg·L^−1^ PLA indicate that fluorescence kinetics around the J step were particularly sensitive to high PLA exposure. However, these fluorescence responses do not identify the J step as the primary molecular site of PLA action.

Taken together, OJIP-derived parameters demonstrate that 200.0 mg·L^−1^ PLA primarily disrupted PSII photochemical function by accelerating the closure of PSII reaction centers and limiting electron transport beyond Q_A_, and the disturbance of the PSII acceptor side provides an early mechanistic explanation for the decline in photosynthetic performance under high PLA exposure and highlights the value of OJIP analysis for resolving the photosynthetic targets of aromatic organic acid stress in cyanobacteria.

### 4.3. L-Band and K-Band Analyses Indicate Impaired Energetic Connectivity of PSII Units and Function of Donor Side in PSII

The OJIP phase encapsulates complex information that may not be readily discernible from the raw fluorescence curve. Consequently, it is common practice to employ double normalization of the transients between specific steps to elucidate hidden bands associated with structural and functional changes in Photosystem II (PSII). The L-band, derived from transients normalized between the O and K steps, indicates energetic connectivity or grouping among PSII units [41,42]. Generally, a positive L-band suggests a reduction in energetic connectivity among the antenna-PSII reaction center units, implying less efficient sharing of excitation energy between adjacent PSII units [42]. In this study, the W_OK_ curves for both the control and low PLA treatment were nearly identical, whereas the 200.0 mg·L^−1^ PLA treatment resulted in a distinctly positive δW_OK_ signal. Furthermore, the observed increases in F_L_/F_J_ and F_J_ indicate that high levels of PLA exposure not only impeded electron transport but also disrupted the energetic coordination between the antenna complexes and the PSII reaction centers.

The K-band, defined by the fluorescence transients normalized between the O and J steps, serves as an indicator of PSII donor-side limitations, particularly highlighting impairments in the oxygen-evolving complex (OEC) [43]. The emergence of the K step, occurring at approximately 300 μs, generally signifies a kinetic imbalance wherein electron donation from the OEC fails to keep pace with electron withdrawal by the acceptor side. In this study, exposure to 200.0 mg·L^−1^ of PLA also induced a significant positive δW_OJ_ signal, an increase in both W_K_ and F_K_/F_J_, and a reduction in the relative abundance of active OEC centers. These findings collectively suggest that electron supply from the donor side becomes insufficient under high PLA exposure. Notably, PLA exposure not only restricts electron movement beyond Q_A_, as indicated by V_J_ and Mo, but also compromises the donor side’s capacity to replenish electrons within the PSII complex. Taken together, the positive L- and K-band responses are consistent with reduced energetic connectivity among PSII units and increased donor-side limitation under high PLA exposure. However, these fluorescence signatures support functional perturbation of PSII, and are not used to identify the underlying structural or molecular lesion.

### 4.4. I–P-Phase Analysis Suggests Altered Electron Transfer Beyond PSII

Chlorophyll *a* fluorescence analysis serves as a robust tool for investigating not only the primary photochemistry of Photosystem II (PSII) but also the subsequent electron transport processes. Within the OJIP transient, the I–P phase is attributed to electron flow beyond the plastoquinone pool and the reduction of electron acceptors on the Photosystem I (PSI) acceptor side [24]. Consequently, the normalization of the fluorescence transient in the O–I and I–P intervals is extensively employed to assess whether a stress factor impacts electron transport beyond Q_A_ and the intersystem electron transport chain. Notably, electron transport in cyanobacteria is not adequately represented by a simple linear PSII-to-PSI sequence. Photosynthetic and respiratory electron flows share the thylakoid membrane and exchange carriers, and cyclic electron flow around PSI, together with terminal oxidases, further modulates the redox poise of the chain, particularly under stress. We therefore caution that I–P phase kinetics and the OJIP-derived parameters derived from them do not, on their own, report exclusively on linear photosynthetic electron transport. In this study, we treat these parameters as readouts of altered downstream electron transfer and redox state, while keeping in view the plausible contributions of respiratory and cyclic pathways. In the current study, the kinetics of W_OI_ and I-P intervals exhibited distinct deviations under the 200.0 mg·L^−1^ PLA treatment compared to the control, indicating a significant disruption in the electron transport sequence between the J and I regions. Meanwhile, the W_IP_ curve was suppressed under high PLA exposure, indicating a restriction in the development of the I–P phase. Given that the I–P phase reflects the reduction of electron carriers in the downstream of PSII, these findings suggest that high PLA exposure disrupted the equilibrium between electron input from PSII and its subsequent consumption or transfer to terminal acceptors.

The quantum yields and efficiencies of electron transport further corroborate this interpretation. Specifically, ETo/TRo represents the efficiency with which a trapped exciton facilitates electron movement beyond QA^−^, while ETo/ABS denotes the quantum yield of electron transport derived from absorbed light energy. A reduction in ETo/TRo and ETo/ABS under exposure to 200.0 mg·L^−1^ PLA indicates that a smaller fraction of trapped excitons or absorbed excitation energy was converted into electron flow. This observation aligns with previous OJIP evidence indicating enhanced accumulation of Q_A_^−^ and premature closure of reaction centers. Consequently, high PLA exposure not only impedes the initial reduction events at the PSII acceptor side but also significantly diminishes the efficiency of electron flow from PSII to the downstream transport chain. Regarding the half-rise time (the time point at which W_IP_ = 0.5), it reflects the reduction rate of the PSI end-electron acceptor pool. An increase in half-rise time values under PLA exposure suggests a decrease in the reduction rates of these acceptors. Interestingly, while the overall I–P phase was restricted, the significant increase in δ_Ro_ indicates that PLA altered the balance of electron transfer between PSII and downstream acceptor-side processes. Meanwhile, direct measurements of PSI activity would be required to determine whether PSI itself undergoes compensatory regulation under PLA exposure.

### 4.5. NPQ Responses Reveal Enhanced Energy Dissipation and Limited Photochemical Recovery

Non-photochemical quenching (NPQ) represents a primary photoprotective mechanism that dissipates excess excitation energy as heat when light absorption surpasses the capacity for photochemical conversion, thereby mitigating the risk of photoinhibition and photodamage [28,44,45]. The parameters of photochemical and non-photochemical quenching, as assessed through chlorophyll fluorescence analysis, provide complementary insights into the balance between the utilization and dissipation of light energy. To enhance our understanding, we also examined the specific energy fluxes per active PSII reaction center. In the current study, we analyzed the distribution of light energy within the PSII reaction center. Under the condition of 200.0 mg·L^−1^ PLA treatment, both ABS/RC and TRo/RC increased, indicating that each active PSII reaction center was subjected to a higher excitation load. However, this did not correspond to a proportional increase in electron transport efficiency (ETo/RC). In this instance, DIo/RC increased significantly, suggesting that a larger fraction of the absorbed and trapped energy was dissipated rather than utilized for electron transport.

The parameters related to light induction further corroborated this interpretation. Specifically, QY denotes the effective quantum yield of PSII under light-adapted conditions, while Qp indicates the proportion of open PSII reaction centers. Additionally, NPQ reflects the non-photochemical dissipation of surplus excitation energy as heat. Under the 200.0 mg·L^−1^ PLA treatment, both QY and Qp consistently exhibited lower values compared to the control throughout the light induction process, whereas NPQ was significantly elevated. These coordinated changes in parameters suggest that high levels of PLA exposure inhibited the efficiency of photochemical energy conversion, leading to the closure of reaction centers.

The increase in NPQ under 200.0 mg·L^−1^ PLA is an indicator of increased non-photochemical energy dissipation in the actinic irradiance tests. Nonetheless, since NPQ was measured at a single, comparatively low light intensity that was equivalent to the cultivation irradiance, this response cannot be evaluated without any ambiguity as an adaptive photoprotective response. The increases in NPQ combined with the decreases in QY and Qp suggest more conservatively that the excitation energy is redistributed at high PLA exposures, and the energy is no longer used to produce photochemical products. It remains to be determined whether this alteration is an active photoprotective effect or a more general effect of actinic light on metabolism, or a combination of both, that should be tested over a wider range of actinic-light levels. It is important to note that the dark-recovery measurements assess short-term recovery following actinic illumination and do not determine whether PLA-induced PSII inhibition is reversible following extended elimination of PLA. To differentiate between transient inhibition or permanent damage, a special recovery experiment with transfer of exposed cells to new PLA-free medium and monitoring over time would be necessary.

Despite several insights obtained in this study, a number of experimental limitations still exist. First, because the pH of the culture medium was not monitored or controlled in a timely manner following PLA addition, the physiological responses of the algal strains should not be interpreted exclusively as PLA-specific chemical toxicity. Further research should incorporate pH-matched controls and pH-adjusted PLA treatments to distinguish between the direct effects of PLA and those associated with medium acidification. Second, additional UV–Vis analysis showed negligible absorbance of PLA around 680 nm, indicating that PLA itself was unlikely to interfere directly with OD_680_ measurements. OD_680_ was used to estimate biomass by using a calibration relationship with dry weight. However, cyanobacterial OD_680_ is affected by both the cellular pigment content and cell abundance; the resulting significant decrease in chlorophyll *a* under 200.0 mg·L^−1^ PLA might have created uncertainties in the biomass estimates. The fact that PLA-treated samples were not confirmed by direct methods of determining biomass (via dry weight) or cell counts suggests that the extent of biomass reduction under the highest PLA concentration must be viewed with some concern. Further research needs to provide an independent estimate of biomass alongside OD-based estimates, especially in situations where there is significant pigment loss. Third, diluting the algal suspension with PLA-free BG11 prior to fluorescence measurements showed that the nominal concentration of PLA was not maintained during the subsequent 30 min dark-adaptation period. Consequently, the fluorescence responses in this study represent the physiological state maintained after 48 h of PLA exposure, rather than responses under continuous PLA exposure during the measurement period. Meanwhile, UV–Vis analysis of cell-free BG11 containing 100.0 and 200.0 mg·L^−1^ PLA showed negligible absorbance around 455 nm, indicating that direct attenuation of the blue excitation light by PLA was unlikely to substantially interfere with the chlorophyll fluorescence measurements. Moreover, the experiments were performed using a single cyanobacterial strain under controlled laboratory conditions, and marked growth inhibition was primarily observed at 200.0 mg·L^−1^ PLA, but it remains uncertain whether such a concentration is feasible in actual water bodies.

Finally, PLA stability was not monitored during the 48 h exposure period. Therefore, possible changes in PLA concentration due to photochemical, hydrolytic, or biological transformation cannot be excluded, and the concentrations reported in our study should be interpreted as nominal rather than analytically verified exposure concentrations. Further, the current research lacked the establishment of persistence or microbial degradation dynamics of PLA in natural waters. Despite the fact that PLA has been known to be involved in microbial metabolic and biotransformation pathways, quantitative data on the degradation rate and half-life of PLA in natural freshwater microbial communities is not adequately characterized. Thus, the current lab experiments cannot be used to draw conclusions about the environmental persistence of PLA. Natural-water microcosm studies, in combination with time-resolved chemical analysis and sterile controls, will be necessary in the future to measure the rates of microbial degradation, transformation products and half-lives of compounds in the environment under various conditions. Meanwhile, the photodegradation kinetics of PLA in ambient sunlight were not considered in the current study, and there is still a paucity of reliable quantitative data on its aqueous photolysis rate and its environmental half-life. Therefore, the survival of PLA during natural solar irradiation cannot be deduced at the moment. Photolysis rate constants, half-lives, and major transformation products under environmental conditions need to be determined by direct measurements under natural sunlight or standardized solar-simulator conditions. Finally, the current study failed to evaluate persistence, biodegradation, transformation products, and ecotoxicological effects of PLA on non-target aquatic organisms. Future studies should evaluate environmentally relevant exposure ranges, degradation kinetics, non-target toxicity, and performance in natural or semi-natural aquatic systems before the practical use of PLA for cyanobacterial bloom control can be considered.

## 5. Conclusions

To sum up, we investigated the effects of various concentrations of PLA on biomass, photosynthetic pigments, and photosynthetic activity in FACHB-82. The findings revealed that PLA induced a concentration-dependent biphasic response on biomass accumulation and photosynthetic pigments of FACHB-82. Chlorophyll *a* fluorescence analyses revealed that high-concentration PLA exposure disrupted functions of PSII at multiple levels. High-concentration PLA exposure enhanced Q_A_^−^ accumulation, indicating accelerated PSII reaction center closure and restricted electron transport beyond Q_A_. L-band and K-band analyses further revealed reduced energetic connectivity among PSII units and impaired donor-side function. I–P-phase and NPQ analyses showed restricted downstream electron transport, enhanced non-photochemical energy dissipation and incomplete recovery of PSII photochemical efficiency after illumination. This study highlights the potential of PLA as an anticyanobacterial compound that warrants further investigation for the control of cyanobacterial blooms. However, our study primarily evaluated the inhibitory effect of PLA against a single cyanobacterial strain under laboratory conditions, and pronounced inhibition was mainly observed at 200 mg·L^−1^ PLA. Further studies are required to evaluate its effective concentration range under environmentally relevant conditions, environmental persistence and biodegradation, potential degradation products, and possible effects on non-target aquatic organisms.

## Data Availability

The original contributions presented in this study are included in the article/Supplementary Material. Further inquiries can be directed to the corresponding authors.

## Supplementary Materials

The following supporting information can be downloaded at: https://www.mdpi.com/article/10.3390/microorganisms14102217/s1, Figure S1: UV–Vis absorption spectra of PLA in cell-free BG11 medium. PLA solutions at 100 and 200 mg·L^−1^ were scanned over 320–750 nm using BG11 medium as the blank. The vertical dashed lines indicate 455 nm, corresponding to the excitation wavelength used for chlorophyll fluorescence measurements, and 680 nm, corresponding to the wavelength used for OD-based biomass estimation. Absorbance values at both wavelengths remained close to zero, indicating negligible direct optical interference from PLA under the tested conditions. Slight negative absorbance values reflect baseline correction and instrumental variation relative to the BG11 blank; Figure S2: Standard curve between algal biomass and OD_680_ values. A strong linear relationship was also observed with a coefficient of determination (R^2^) value of 0.9938; Figure S3: Enlarged W_OI_ curves in the W_OI_ ≥ 1 region, showing the trend of the I–P phase; Figure S4: Energy flux parameters per reaction center, including ABS/RC, TRo/RC, ETo/RC and DIo/RC. Values represent the mean of three independent measurements, and bars indicate SD. Different letters indicate a significant difference at the 0.05 level (*p* < 0.05, Duncan’s multiple range test); Table S1: Definitions of selected chlorophyll fluorescence and JIP-test parameters.
