# Supplementary material for: Phenyllactic Acid Suppresses Growth and Induces Coordinated Changes in PSII Photochemistry and Energy Dissipation in the Filamentous Cyanobacterium Dolichospermum sp. FACHB-82
Source: Microorganisms. 2026 Oct 2;14(10):2217. doi: 10.3390/microorganisms14102217 (PMC13707886; doi:10.3390/microorganisms14102217)
Supplement: Supplementary file 1 [file microorganisms-14-02217-s001.zip › microorganisms-4572829-supplementary.pdf]

Supporting information

Phenyllactic Acid Suppresses Growth and Induces Coordinated Changes in PSII Photochemistry and Energy Dissipation in the Filamentous Cyanobacterium *Dolichospermum* sp. FACHB-82

Jie Cheng [1], Long Tan [1], Xiu Zhang [1], Zhenxia Zhu [1], Jiashun Li [2,*], Chaobo Zhang [1], Kaidian Zhang [2] and Yongcheng Ding [1,3,*]

[1] Shandong Key Laboratory of Applied Technology for Protein and Peptide Drugs, School of Pharmaceutical Sciences and Food Engineering, Liaocheng University, Liaocheng 252000, China; chengjie@lcu.edu.cn (J.C.); tldyp6789@163.com (L.T.); 15216501768@163.com (X.Z.); 17661802272@163.com (Z.Z.); zhangchaobo@lcu.edu.cn (C.Z.)

[2] State Key Laboratory of Marine Resources Utilization in South China Sea, School of Marine Sciences, Hainan University, Haikou 570100, China; kzhang@hainanu.edu.cn

[3] Shanghai Academy of Environmental Sciences, Shanghai 200233, China

* Correspondence: jiashun.li@hainanu.edu.cn (J.L.); ycding@smail.nju.edu.cn (Y.D.)

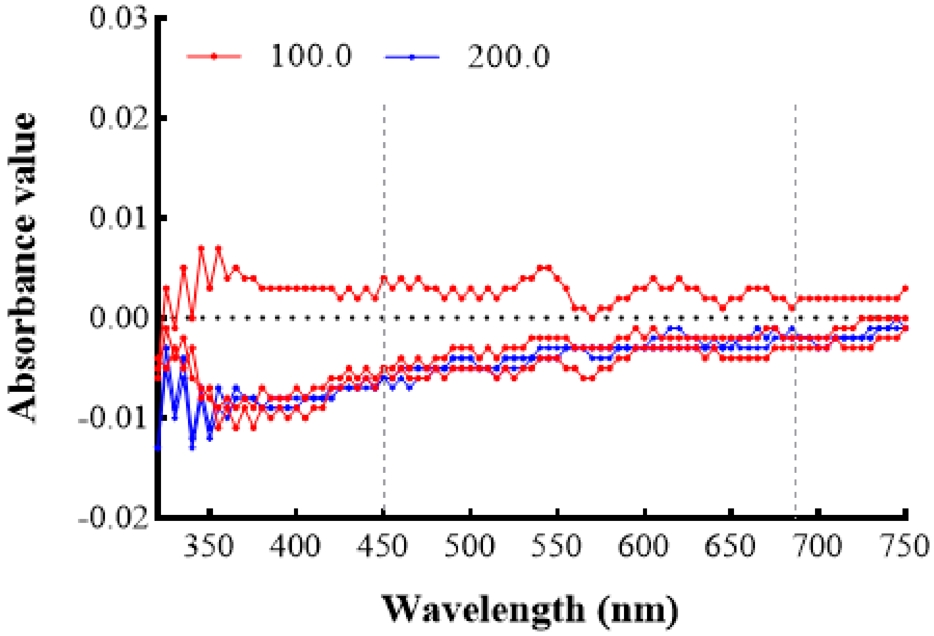

Figure S1 UV-Vis absorption spectra of PLA in cell-free BG11 medium. PLA solutions at 100 and 200 mg·L^{-1} were scanned over 320-750 nm using BG11 medium as the blank. The vertical dashed lines indicate 455 nm, corresponding to the excitation wavelength used for chlorophyll fluorescence measurements, and 680 nm, corresponding to the wavelength used for OD-based biomass estimation. Absorbance values at both wavelengths remained close to zero, indicating negligible direct optical interference from PLA under the tested conditions. Slight negative absorbance values reflect baseline correction and instrumental variation relative to the BG11 blank.

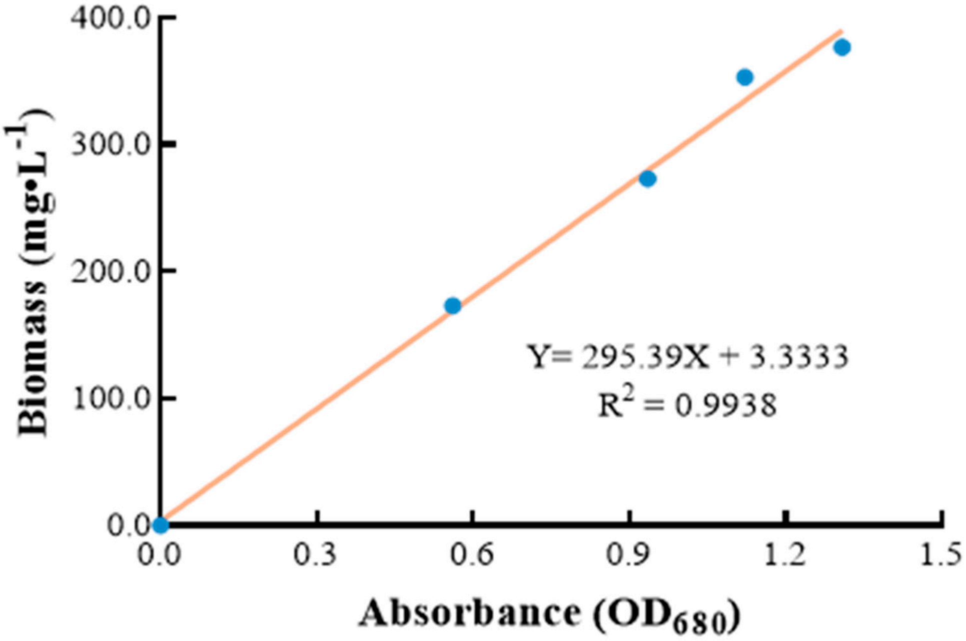

Figure S2 Standard curve between algal biomass and OD_{680} values. A strong linear relationship was also observed with a coefficient of determination (R^2) value of 0.9938.

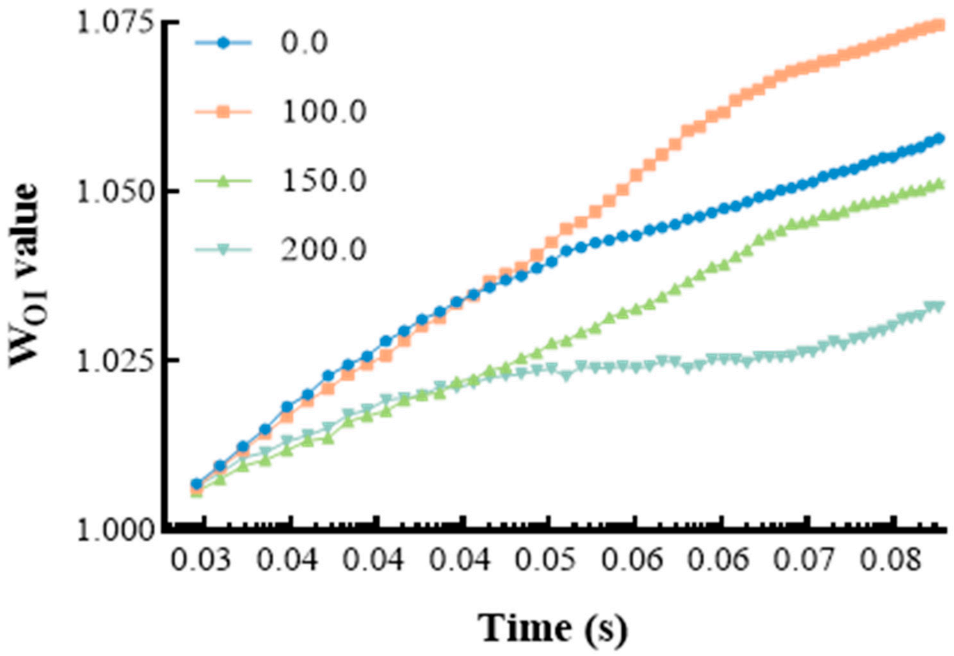

Figure S3 Enlarged W_{OI} curves in the $W_{OI}\geq 1$ region, showing the trend of the IP phase.

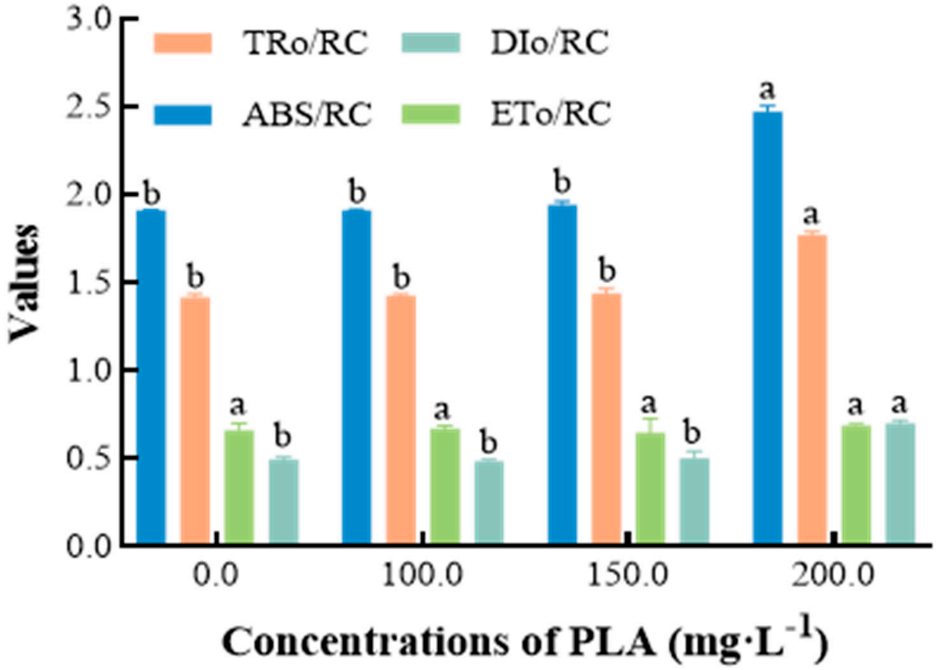

Figure S4 Energy flux parameters per reaction centre, including ABS/RC, TRo/RC, ETo/RC and DIo/RC. Values represent the mean of three independent measurements and bars indicate SD. Different letters indicate a significant difference at the 0.05 level ($P<0.05$, Duncan's multiple range test).

Table S1 Definitions of selected chlorophyll fluorescence and JIP-test parameters.

JIP-test	Information note
Fo	Minimal fluorescence intensity
F_K	Fluorescence intensity at 0.3 ms
F_L	Fluorescence intensity at 0.15 ms
F_J	Fluorescence intensity at 2 ms
F_I	Fluorescence intensity at 30 ms
Fm	Maximal fluorescence intensity
V_J	Relative variable fluorescence intensity at the J-step
V_I	Relative variable fluorescence intensity at the I-step
Fv/Fm	Maximum quantum yield of PSII
PIabs	Performance index on absorption basis
Mo	Approximated initial slope of relative variable fluorescence
Sm	Normalized total complementary area above the OJIP curve
ABS/RC	Absorption flux per reaction center
TRo/RC	Trapped energy flux per reaction center
ETo/RC	Electron transport flux per reaction center
DIo/RC	Dissipated energy flux per reaction center
ETo/TRo	The probability that a trapped exciton moves an electron into the electron transport chain beyond Q_A
ETo/ABS	Quantum yield for electron transport
W_K	The damage degree of the donor side of PSII reaction center
OEC centers	The active fraction of oxygen evolving complexes centers
δ_{Ro}	The probability that an electron from the intersystem electron carriers to reduce end electron acceptors at the PSI acceptor side
